# OptMAVEn – A New Framework for the *de novo* Design of Antibody Variable Region Models Targeting Specific Antigen Epitopes

**DOI:** 10.1371/journal.pone.0105954

**Published:** 2014-08-25

**Authors:** Tong Li, Robert J. Pantazes, Costas D. Maranas

**Affiliations:** Chemical Engineering Department, The Pennsylvania State University, University Park, Pennsylvania, United States of America; Technical University of Braunschweig, Germany

## Abstract

Antibody-based therapeutics provides novel and efficacious treatments for a number of diseases. Traditional experimental approaches for designing therapeutic antibodies rely on raising antibodies against a target antigen in an immunized animal or directed evolution of antibodies with low affinity for the desired antigen. However, these methods remain time consuming, cannot target a specific epitope and do not lead to broad design principles informing other studies. Computational design methods can overcome some of these limitations by using biophysics models to rationally select antibody parts that maximize affinity for a target antigen epitope. This has been addressed to some extend by OptCDR for the design of complementary determining regions. Here, we extend this earlier contribution by addressing the *de novo* design of a model of the entire antibody variable region against a given antigen epitope while safeguarding for immunogenicity (Optimal Method for Antibody Variable region Engineering, OptMAVEn). OptMAVEn simulates *in silico* the *in vivo* steps of antibody generation and evolution, and is capable of capturing the critical structural features responsible for affinity maturation of antibodies. In addition, a humanization procedure was developed and incorporated into OptMAVEn to minimize the potential immunogenicity of the designed antibody models. As case studies, OptMAVEn was applied to design models of neutralizing antibodies targeting influenza hemagglutinin and HIV gp120. For both HA and gp120, novel computational antibody models with numerous interactions with their target epitopes were generated. The observed rates of mutations and types of amino acid changes during *in silico* affinity maturation are consistent with what has been observed during *in vivo* affinity maturation. The results demonstrate that OptMAVEn can efficiently generate diverse computational antibody models with both optimized binding affinity to antigens and reduced immunogenicity.

## Introduction

Therapeutic antibodies are widely recognized to be among the most promising agents to treat various diseases, including cancers, immune disorders, and infections [1], [2]. The earliest used technology for the generation of therapeutic antibodies is raising antibodies against a target antigen in immunized mice. Although widely utilized, the low clinical success rate using mouse antibodies reflects that these foreign proteins can be highly immunogenic in humans, and they typically have weak interactions with human complement and antibody receptors, resulting in inefficient effector functions [3]. These limitations have largely been overcome by grafting the variable domains of a mouse monoclonal antibody to the constant domains of a human antibody, a process known as chimerization [4], [5]. Although chimeric antibodies are more human-like and induce considerably less response by the human immune system, they are still not completely human. More recently, complete human antibodies have been designed using directed evolution techniques [6], [7] that mimic the natural selection of the process to evolve antibodies towards a desired property. Among them, phage display [8], [9], a technique based on the presentation of peptides or protein fragments on the surface of bacteriophages, is most widely used and offers robust and complementary routes to the generation of potent human antibodies. Despite these advances in the design of antibodies, current experimental methods still have considerable limitations and cannot: (1) target a specific antigen epitope, (2) provide universally applicable structural design routes, and (3) rationally engineer mutations with significantly reduced immunogenicity.

By contrast, computational methods could efficiently overcome some of these shortcomings. For example, a number of successful applications of computational methods have been reported in antibody-antigen recognition [10]–[13], antibody structure and stability prediction [14]–[17], design of mutations and antibody-antigen interface [16]–[22], and immunogenicity prediction [23], [24]. However, most of the current examples of computational antibody design have been largely limited to existing antigen–antibody complex structures (i.e. re-designs of antigen–antibody interfaces), and the *de novo* design of antibodies to target a pre-selected antigen epitope has remained elusive.

**Figure 1:**
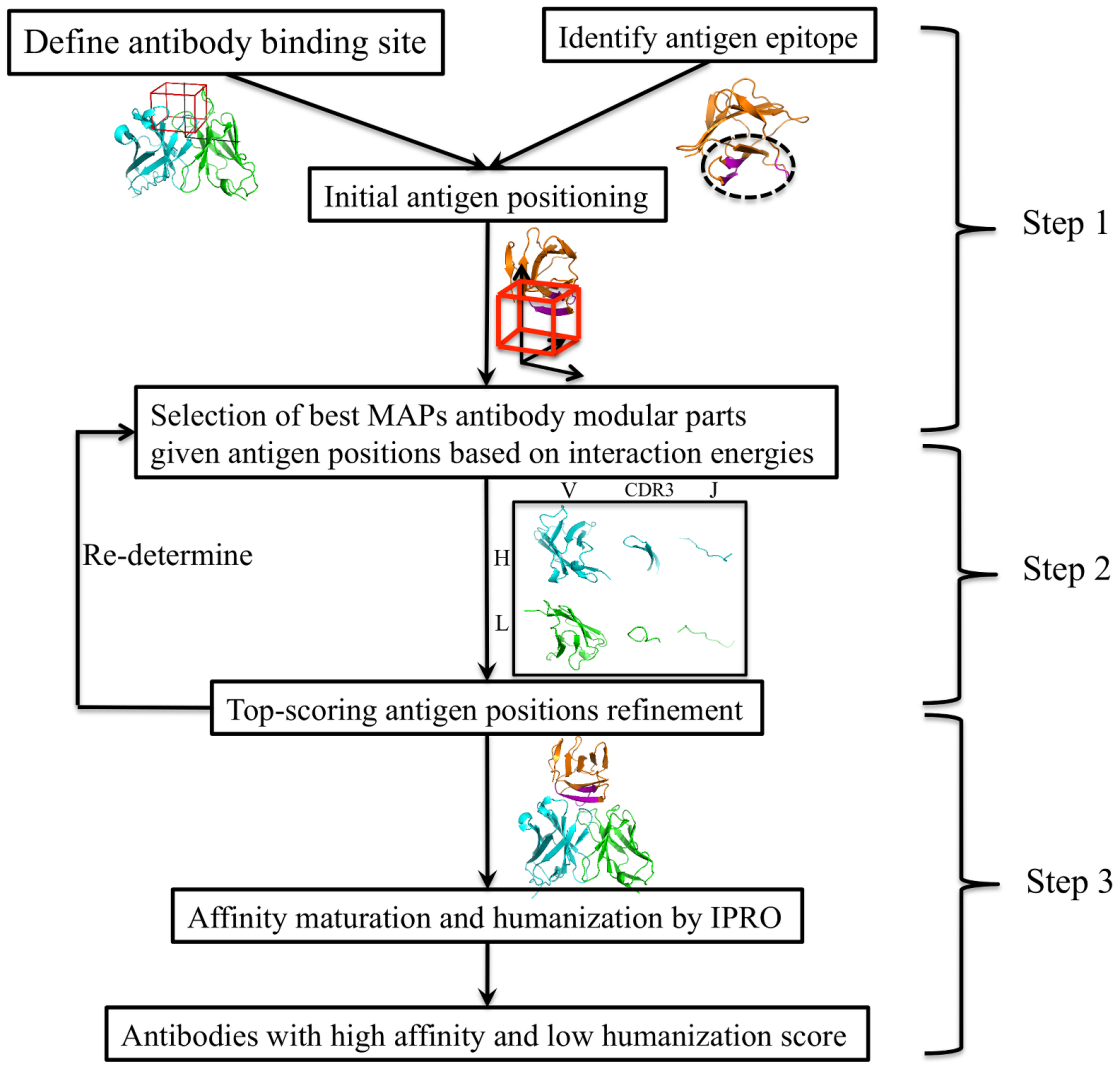
OptMAVEn workflow. Step 1: Antigen positioning. Step 2: Assembly of antibody models targeting the antigen epitope. Step 3: Affinity maturation and humanization of antibody models by redesigning.

**Figure 2:**
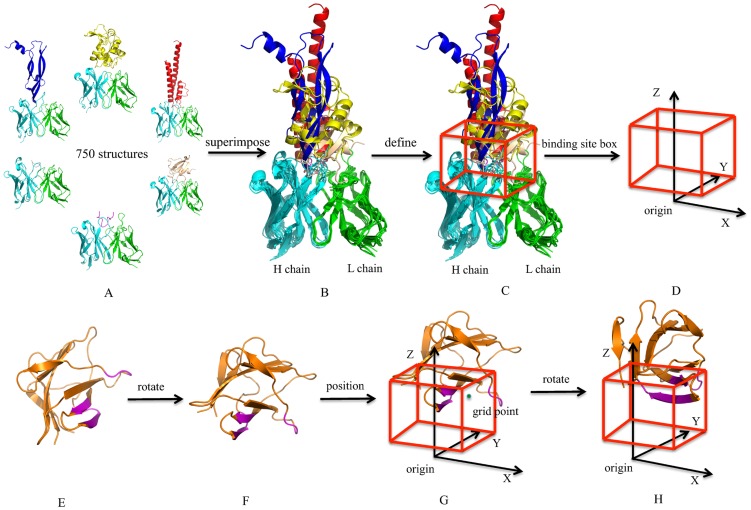
Illustrations of antibody-binding site and the algorithm of antigen positioning. H and L chains are colored in cyan and green, respectively; epitope is colored in magenta. (A) Database of 750 antibody-antigen structures. H and L chains are colored in cyan and green. Antigens are in different colors. (B) All the complex structures superimposed onto a reference antibody structure whose coordinate center of CDRs attachment points was placed on the origin. (C) A rectangular box that covers all the mean epitope coordinates. Figure S2 shows the distributions for the mean coordinates of all the epitopes along the X, Y, and Z axes. (D) The virtual antibody-binding site. (E) An antigen initial conformation. Epitope is colored in magenta. (F) The rotated antigen conformation having the most negative epitope coordinates. (G) A positioned antigen conformation with epitope's geometry center at one grid point. (H) A rotated antigen conformation around Z axis.

To address the limitations of current platforms for antibody design, we have developed the OptCDR method that can *de novo* design an antibody paratope model against any targeted antigen epitope by modeling and optimizing the complementarity determining regions (CDRs) [21]. However, CDRs only capture part of the binding capacity of an antibody and were not constrained to fully human designs. Therefore, in this paper we take the next step and introduce a new computational framework named OptMAVEn for *de novo* design of not just the CDRs, but fully human, complete antibody variable domain models by expanding the concepts pioneered in OptCDR. OptMAVEn designs antibody models by mimicking the natural evolution of antibody generation and affinity maturation (Fig. S1). In particular, it is implemented as a three-step workflow (Fig. 1). First, for a given antigen, an ensemble of possible antigen binding conformations is generated in a virtual antibody-binding site. This site is defined as a rectangular box that covers all the geometry centers of 750 antigen epitopes with known structures (Fig. 2A–2D). Second, the best scored antigen conformation and combination of six modular antibody parts from the Modular Antibody Parts (MAPs) database [25] are selected using a mixed-integer linear programming (MILP) formulation and the initial antibody structure is predicted by assembling the six MAPs. Third, the antibody model is redesigned and optimized through our Iterative Protein Redesign & Optimization (IPRO) protocol [26].

A new aspect in the last step of OptMAVEn is that we incorporated a 9-mer-sequence-based humanization algorithm to reduce the potent immunogenicity of designed antibody models while optimizing their binding affinity to an antigen. The immunogenicity of an antibody is triggered by molecular recognition of its immunogenic peptides by the major histocompatibility complex (MHC) and/or T cells [27]. A variety of humanization methods such as CDR grafting [28], resurfacing [29], superhumanization [30], framework shuffling [31] and guided selection [32] have produced antibodies more homologous to human sequences, often but not always leading to reductions in immunogenicity to clinically acceptable levels [33]. Our approach is inspired by a humanness score termed “human string content” (HSC) [23] that quantifies a sequence at the level of potential MHC/T cell epitopes. HSC is principally based on the assumption that having more human sequence present in the antibody will reduce its immunogenic potential [34]–[36].

OptMAVEn aims at designing a diverse library of antibody models that is simultaneously co-optimized on both binding affinity to an antigen epitope and immunogenicity. The resulting computational antibody models are *de novo* designs, probably never seen in nature before, generated *de novo* through computations. In this study, we benchmarked the corresponding protocols in each step individually and show that OptMAVEn could: (1) efficiently position antigens in the antibody-binding site with a high success rate of 96% using a benchmark set of 120 experimentally determined antigen-antibody complexes (2) rediscover 57.5% of native antibody parts using MILP based optimization for the same benchmark set (3) recapitulate known interactions responsible for affinity maturation using two known germline and affinity maturation antibody pairs as the benchmark set and (4) unambiguously distinguish human antibody sequences from other species (P value <0.000001). As case studies, we applied OptMAVEn to design broadly neutralizing antibody models against two antigens: envelope glycoprotein gp120 (gp120) and hemagglutinin (HA), which are well-known antigens for the HIV-1 and influenza viruses, respectively. The presented designs are diverse in sequence and structure spanning a wide array of affinity maximization interactions. These designed interactions (partially) mimic the geometry of their natural receptors while maintaining the computational humanness of the sequences. Overall, our results demonstrate that OptMAVEn is a computational framework for an open challenge that could make significant contribution to the development of a new generation of therapeutic antibodies and vaccines.

## Method

### Step 1: Antigen positioning

Antigen positioning starts with the definition of a general antibody-binding site (Fig. 2A–2D), which is represented by a rectangle grid box located close to the origin. This box was obtained by analyzing the locations of known antigen epitopes from a precompiled antibody-antigen crystal structure database consisting of 750 antibody-antigen complexes and sharing three common features: (1) X-ray resolution is better than 2.5 Å, (2) both heavy and light chains are available in the structures, and (3) an antigen is included for each structure. Among the 750 structures, there are 214 hapten, 109 peptide and 427 protein binders. The size of the box was adjusted to include all the mean coordinates of atoms of antigen epitopes, and its X, Y and Z values were within the ranges of (−10 Å, 5 Å), (−5 Å, 10 Å) and (3.75 Å, 16.25 Å), respectively (Fig. 2D). The box was divided into a set of grid points by assigning grid spacing, which is user-defined (default of 2.5, 2.5 and 1.25 Å for X, Y and Z axis, respectively). During the positioning, the coordinate center of an antigen epitope was placed into its corresponding grid box (Fig. 2G) and rotated along the Z coordinate for 360° with an interval of 60° (Fig. 2H) to generate an ensemble of initial antigen positions. Prior to positioning, the antigen was rotated so that the epitope had the most negative Z coordinates (Fig. 2E and 2F), thus ensuring that the target epitope forms the maximum number of interactions with the CDRs. In total, 3234 (7×7×11×6) initial positions of antigens were generated for this study.

### Step 2: Assembly of antibody models targeting the antigen epitope

At each position, the interaction energies (IEs), including van der Waals and electrostatic terms, between the antigen and all MAPs were calculated and stored. To avoid detrimental clashes, a “softening” atom van der Waals radius whose value is half of that from the CHARMM force field [37] was used to estimate the hydrophobic interaction. The modular antibody parts were previously constructed in the spirit of template-based modeling, with each part being a prototype structure of the random variable (V), diversity (D), and joining (J) regions in the MAPs database [25]. The recently generated database contains 929 parts constructed from an analysis of 1168 human, humanized, chimeric, and mouse antibody structures and encompasses all currently observed structural diversity of antibodies. Table S1 shows the numbers of antibody parts from the MAPs database. V, CDR3 and J structures can assemble both H and L chains of an antibody. There exist two types of light chain, KAPPA and LAMBDA, which are treated separately. Each MAPs structure was numbered using IMGT's unique numbering [38]–[41] and consistently placed in the 3D space so that its CDRs attachment points were approximately on the same X-Y plane and centered on the origin with CDRs perpendicularly directed in the positive Z direction.

Once the IEs are calculated, the problem of selecting the best scoring combination of non-clashing antibody parts at each position could be mathematically represented using an MILP representation. This requires the definition of the index set *I* 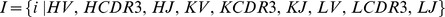, denoting modular antibody parts. H denotes Heavy, K for KAPPA and L for LAMBDA. V denotes Variable region, CDR3 for Diversity region and J for the Joining region. Also required is the set 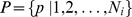, which denotes the number of MAPs structures at position *i*. Set 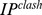 contains all pairwise MAPs combinations (*i*1, *p*1) and (*i*2, *p*2) that sterically clash with one another. The binary variables 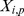 denotes if antibody part *p* is selected at position *i* (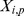 = 1) or not (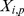 = 0). Parameter 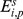 encodes the calculated energy between structure *p* at position *i* and the antigen substrate. Switching parameters *H_d_* and *L_d_* have values of one if a VH or VL respectively, are being designed and zero if they are not chosen. This allows the user to possibly design only a VH or VL, as would be appropriate for a nanobody. The MILP formulation can then be written as follows:
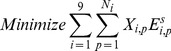
(1)

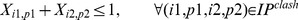
(2)

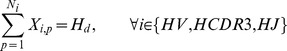
(3)

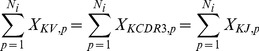
(4)

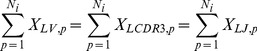
(5)

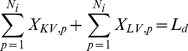
(6)


Equation 1, the objective function, entails the minimization of the interaction energy between the antigen and the selected MAPs. The inequality in Equation 2 precludes the simultaneous presence of two antibody part structures that sterically clash. Equation 3 guarantees exactly one part is selected for each heavy chain region when a VH is designed. OptMAVEn defines both KAPPA and LAMBDA light chains and Equations 4–6 make sure that when a VL is being designed, it is composed of parts entirely from one domain type or the other. Equation 4 requires that the same number of KAPPA V, CDR3, and J structures are selected and Equation 5 does the same for the LAMBDA structures. Equation 6 then selects *either* a KAPPA or LAMBDA V part when a VL domain is being designed, and Equations 4 and 5 together ensure the selection of the proper type of CDR3 and J structures. The optimization formulation described collectively by Equations (1–6) can be solved using CPLEX [42] called directly from Python.

After the MILP selection, the lowest energy solutions (10 in our current study) were selected and submitted to local refinement (i.e., 500 iterations) by randomly moving antigens and reselecting the optimal MAPs. The goal of the refinement is to explore the local conformational space for the antigen and to energetically rescreen the best MAPs. In each iteration, the antigen was randomly translated and rotated for a small distance and angles whose value was generated using a Gaussian distribution centered at zero with a standard deviation of 0.5 Å and 5°, respectively. Subsequently, the IEs between the antigen at this position and the entire MAPs database were reevaluated. A simulated annealing algorithm was used to determine whether or not to retain the results of the iteration.

### Step 3: Computational affinity maturation and humanization by redesigning

The refined antibody models were redesigned with IPRO [26] in order to find sequences that maximally improve the binding affinity and possess minimal computational immunogenicity. The standard IPRO design protocol was modified for use in OptMAVEn, which consists of five main steps: sequence design, backbone perturbation, optimal rotamer selection, antigen redocking and energy evaluation. This sequence is carried out iteratively (default max of 10,000 iterations).

#### Sequence design

For each round, a set of 1–3 continuous residues in either VH or VL is randomly selected for mutation. Residues from CDRs are given 3-fold higher preference over those from frameworks (FRs) because more mutations are expected in the binding site [25]. A selected residue is allowed to mutate to a set of permitted amino acid types that is site-specific and could be determined according to sequence alignments of relevant antibodies. In this study, the alignments of broadly neutralizing HIV and influenza antibody sequences were used (Data S1 and S2).

At the same time, during the sequence design, a sequence-based humanization algorithm is introduced to guarantee that designed sequences are human. This algorithm starts with constructing a human antibody 9-mer sequences database by splitting 69,032 human antibody sequences (collected from Abysis [43], IMGT [38] and the PDB database [44]) into 1,309,657 unique human 9-mers. For a given test sequence, all the possible 9-mer sequence frames are extracted and the minimum number of mutations between each frame and those from the human 9-mer-sequence database is counted. All frame scores are totaled and defined as the humanness score (HScore, Equation 7). 
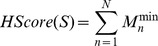
(7)


S denotes a given antibody sequence, N is the total number of all the possible 9-mers-fragment sequences, and M is the minimum count of the mutations between a 9-mer sequence and those from the human 9-mer fragment database. During each iteration only sequences with HScore equal to or lower than the previous iteration are retained for further optimization.

#### Backbone perturbation

Once the sequence is designed, the perturbed region, which includes 5 more residues on both sides of the design positions and surrounding residues within 4.5 Å, is subjected to backbone perturbation. For each one of the perturbed residues, the φ and ψ dihedral angles are randomly perturbed using a Gaussian distribution centered at zero degrees with a standard deviation of 1.5°. No modification greater in magnitude than five degrees is permitted. Five residues on each side of the perturbed region are free to move during the perturbation to prevent the dihedral angle changes from causing long-range structural effects. These residues are mutated to glycine and an energy minimization with strong restraints on the perturbed dihedral angles is carried out to make the perturbed structure. All other residues are fixed in place during the process. Different backbone conformations are sampled by iteratively perturbing small regions of the backbone that are randomly chosen during each cycle throughout the variable domains.

#### Optimal rotamer selection

The following step of IPRO involves the use of a rotamer library and MILP optimization to repack the amino acid side chains in and around the perturbed region. The perturbed residues that are mutated to glycine are always repacked. The residues that are spatially close to the perturbed region are also repacked. Only design positions within the perturbed region are permitted to mutate. The permitted kinds of amino acid mutations for each design position are specified during the sequence design stage. We use the abbreviations R and NR to refer to antibody designed residues (side-chains) and antigen/conserved antibody parts, respectively.

The R–NR (i.e. the antigen and all parts of the antibody that are not being replaced by rotamers) and R-R interaction energies are calculated using the pairwise additive, non-bonded energy terms from the force field (i.e. van der Waals, electrostatics, and implicit solvation). Once all of the R-NR and R-R interaction energies have been calculated, MILP optimization is used to select the minimum energy arrangement of rotamers. The rotamer selection MILP has been previously discussed [26].

#### Antigen redocking

The fourth step of an IPRO iteration is a local, rigid-body redocking of the antigens. Since docking may take a long time for some antigens, this step is only carried out every third iteration. Docking uses the same pairwise additive energy functions used during the rotamer selection step. During docking, an antigen is randomly perturbed along and around the X, Y, and Z axes. The perturbations are generated using a Gaussian distribution centered at zero, with user-defined standard deviations (defaults of 0.2 Å and 2.0°). After the antigen is perturbed, the net IE is evaluated and the movement of the antigen is kept or rejected based on the Metropolis criterion. Each antigen is sequentially randomly perturbed during an iteration of docking, and a user-defined number of iterations are carried out (default of 500 iterations).

#### Energy evaluation

The fifth step involves a total energy minimization and complex and interaction energies evaluation. A high-resolution score function that evaluates van der Waals, electrostatics, bonds, angles, dihedral angles, improper dihedral angles, and generalized Born with molecular volume integration implicit solvation energy functions from CHARMM is used. The complex and interaction energies are used in the Metropolis criterion to determine whether or not to retain the results of the IPRO iteration. The user may set the temperature used to make the decision (default is to retain 25% of designs within 10 kcal/mol of the best design).

### Benchmark test set

For benchmarking our antigen positioning and MILP selection algorithms, we curated a non-redundant set of bound antibody-antigen complex structures gathered from IMGT [40]. Identical antigens in different binding modes with antibodies were also included. This set includes 120 antibody-antigen complex crystal structures (Table S2) and at present, we only focus on the antigens that are either peptides or small proteins. Amino acid lengths for the antigens range from 4 to 148. This set is used to characterize the distribution of antigen epitopes, verify the initial antigen positioning and adjust the energy function. To test the IPRO design algorithm, we chose two germline (GL) / affinity matured (AM) pairs, all with known X-ray structures. Among them, one antibody pair is broadly neutralizing influenza virus antibody CH65 (AM) [45] and its putative GL precursor [16], and another is broadly neutralizing HIV-1 gp120 antibody VRC01 (AM) [46] and its GL precursor [17]. The influenza HA1 and HIV-1 gp120 antigens were both modeled into the binding sites of their corresponding GL antibodies referencing their positions in the AM complex structures. For testing the proposed HScore, human, mouse, rat and rabbit antibody sequences were collected from Abysis [43] with the number of residues between 90 and 130.

### Implementation

OptMAVEn is primarily written in Python and C++ and is available for download on our website, http://maranas.che.psu.edu. The most computationally demanding modules such as energy calculation, antigen positioning and humanness scores calculation are implemented in C++. Parts of OprMAVEn such as energy calculation between antigens and the entire MAPs and computational affinity maturation may use several processors at a time by sharing files. Using its default parameters and 10 processors as an example, a design run against an antigen with 100 amino acids is expected to take no more than three weeks.

## Results

### Benchmark test of the initial antigen positioning

The starting point for an OptMAVEn design is the positioning of the target antigen in a predefined antibody-binding site in order to obtain the initial antigen conformations. To evaluate whether the positioning protocol is capable of generating near-native poses, we tested it against a benchmark set (Table S2) that contains 120 antigens varying in both length and conformations. As described in the Methods and shown in Fig. 2, each antigen was first rotated to make the epitope have the most negative Z coordinates and then positioned into the binding site box with its mean epitope coordinate placed in every grid point with rotations around Z axis. We used Root Mean Squared Deviation (RMSD) of backbone atoms between an antigen position and its corresponding native conformation as a metric for evaluating the quality of positioning. The quality of a positioning is classified as high (RMSD <1.0 Å), medium (1.0 Å<RMSD<2.0 Å), and acceptable (2.0 Å<RMSD<4.0 Å) according to CAPRI-defined criteria for protein docking [11]. A successful prediction was described as a positioning run in which at least one of the ensemble members of initial positions has an acceptable RMSD (i.e.<4.0 Å). The benchmark results of antigen positioning are summarized in Table 1. Across the entire benchmark set, OptMAVEn successfully predicted at least acceptable or better quality solutions for 115 targets, representing an overall success rate of 96%, which indicates OptMAVEn could sample the correct antigen orientation and position without any *a priori* knowledge of antibody-binding site. With respect to antigen type, OptMAVEn successfully predicted near-native structures for 99% of the peptide antigens and 92% of the protein antigens. The slightly better predictions of conformations of peptide antigens are most likely due to their relatively simple and linear epitopes.

**Table 1:** Minimum RMSDs between the native and positioned poses.

Peptide				Protein			
PDB	RMSD	PDB	RMSD	PDB	RMSD	PDB	RMSD
1ACY	1.79	2G5B	1.86	1BJ1	2.11	3DVG	2.71
1CE1	2.35	2H1P	2.98	1DZB	1.35	3E8U	2.40
1CFS	2.41	2HFG	2.50	1EGJ	3.74	3ETB	2.35
1CFT	1.93	2HH0	2.30	1FBI	2.76	3G6D	3.23
1CU4	1.99	2HKF	2.17	1I9R	2.48	3IU3	2.67
1E4W	2.35	2HRP	1.69	1JHL	1.55	3KS0	2.31
1EJO	1.47	2IGF	2.48	1JRH	4.68	3L5W	1.80
1F90	1.69	2J4W	1.83	1KIQ	1.86	3L5Y	1.96
1FPT	1.61	2OQJ	2.62	1MLC	3.42	3LQA	3.88
1GGI	2.43	2OR9	1.79	1NSN	2.12	3NFP	3.09
1HH6	2.02	2OSL	2.85	1OAZ	2.28	3NH7	2.96
1HIN	1.86	2QHR	2.77	1ORS	3.23	3O0R	4.05
1I8I	1.89	2QSC	2.79	1RJL	1.84	3P30	3.79
1JP5	1.86	2R0W	2.02	1TQB	2.23	3QWO	1.97
1KC5	2.06	2ZPK	2.19	1TZH	3.89	3RKD	3.24
1KCR	3.08	3BAE	1.75	1TZI	3.88		
1KTR	2.13	3BKY	2.61	1V7M	2.82		
1MVU	1.28	3CVH	3.13	1W72	2.69		
1N64	1.55	3CXD	2.56	1XIW	2.10		
1NAK	2.57	3EYU	2.42	1ZTX	1.96		
1OBE	4.05	3F58	2.50	2BDN	1.77		
1P4B	2.70	3FFD	2.78	2DQJ	2.03		
1PZ5	2.31	3FN0	2.64	2FJH	3.75		
1QKZ	2.03	3G5V	1.75	2HVK	1.44		
1QNZ	2.27	3GGW	2.24	2IFF	3.52		
1SM3	2.36	3GHB	1.92	2JEL	2.74		
1TET	2.56	3GHE	1.56	2QR0	4.28		
1TZG	1.69	3GJG	2.91	2R29	2.28		
1U8J	2.17	3HR5	2.27	2VWE	4.30		
1UWX	1.74	3MLR	2.16	2VXQ	1.88		
1XGY	2.05	3MLS	1.88	2VXS	3.52		
2A6I	2.33	3MLW	2.37	2ZUQ	2.49		
2B1H	2.14	3MLX	2.08	3AB0	2.36		
2CK0	2.82	3MLY	2.48	3BDY	2.94		
2EH8	2.35	3QG6	2.48	3D85	2.63		
							
	Average	Std	Min	Max	Medium	Accepted	Failure
Peptide	2.25	0.47	1.28	4.05	30%	69%	1%
Protein	2.75	0.84	1.35	4.68	20%	72%	8%

**Figure 3:**
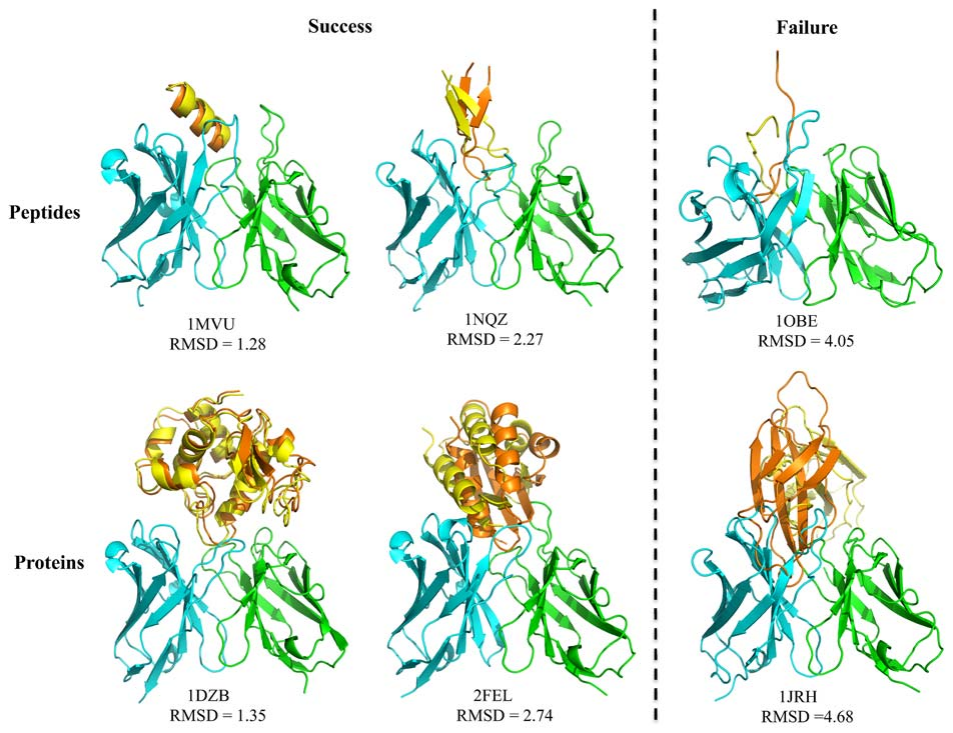
Examples of positioning successes and failures for peptide and protein binders. H and L chains are colored in cyan and green, respectively. Antigens are colored in yellow (native poses) and orange (best positioned poses).

Four representative positioning successes of PDB 1MVU, 1NQZ, 1DZB and 2FEL with RMSDs of 1.28, 2.27, 1.35 and 2.74 Å, respectively, are illustrated in Figure 3. As observed, the predicted positions of 1MVU and 1DZB are in close agreement with their corresponding native poses. For 1NQZ and 2FEL, the prediction quality is considered acceptable and further translations and rotations could improve the positions. The predictions for 1OBE and 1JRH serve as representative examples of positioning failures with RMSDs of 4.05 and 4.68, respectively. For 1OBE, the predicted conformation of its L-shaped antigen adopted an almost opposite orientation compared to its native pose; for 1JRH, considerable differences are derived from rotations around the X and Y axes. In both cases, erroneous positioning mainly arise from the approximation of using coordinate centers of antigen epitopes for representing the entire epitopes, and further rigid-body rotations around X and Y axes are required for successful positioning. Extra rotation sampling around the X and Y axes will almost certainly give more accurate predictions, at the expense of longer computational time.

### Benchmark test for the MILP based residue design step

In OptMAVEn, the variable domain of an antibody structure is initially predicted by assembling the six best-scoring MAPs (HV, HCDR3 and HJ for VH; KV, KCDR3, KJ or LV, LCDR3, LJ for VL) if both the VH and VL are being designed. For a given antigen conformation, the IE between this antigen and each MAPs is calculated using a pairwise energy functions involving both van der Waals and electrostatic terms (MILP energy). The MILP formulation described in Equations 1–6 is used to select the combination of MAPs structures with the lowest IE. To measure whether the MILP selection could identify native antibody parts efficiently, we tested it on the same benchmark set used to evaluate antigen positioning. Starting with only the native pose of each antigen, the IE to the best MAPs is compared to that to its native antibody part. The results are summarized in Table S3. For each complex, we also performed a further energy minimization and reevaluated the IE using the full CHARMM energy function.

In the majority of cases, calculated MILP IEs were negative, indicating favorable binding between antibodies and antigens. In contrast, for five cases (1KTR, 1OAZ, 1TZH, 2A6I and 2IFF) native antibodies were predicted to disfavor the antigen binding. This could arise from subtle steric clashes or over-approximated MILP energy functions. Therefore, we further minimized each complex and reevaluated the IEs using the full Charmm energy function, including the van der Waals, electrostatics, bonds, angles, dihedral angles, improper dihedral angles and generalized Born with molecular volume integration implicit solvation terms. Using this more detailed description for IE led to negative IEs for all antigen-antibody complexes. This suggests that with a relaxation and energy reevaluation step, it is possible for the MAPs structure selection MILP to design initial antibody models that successfully bind the antigen for favorable initial antigen positions (e.g. native positions).

Of particular note is the fact that for 51/120 cases (i.e. 42.5%), the selected antibody models have better CHARMM IEs than the native antibodies, by an average of 95.2±90.7 kcal/mol. In the other 69 cases, the native complex is better by an average of 91.8±64.4 kcal/mol. It is not surprising that OptMAVEn identifies initial antibody models in some cases that have higher energy interaction scores than the native antibodies for two reasons. First, at any given moment the immune system will not have all possible naïve antibodies available, so suboptimal antibodies can be expected to be chosen in many cases. In contrast, MILP selection will always be able to pick an optimal combination of MAPs structures. Second, the objective of an immune response is to eliminate a problem as rapidly as possible, not to design optimally binding antibodies. Although strong binding is important, other factors such as stability, concentration, and when an antibody is discovered play a role. Since OptMAVEn is solely trying to design optimal binders, it is to be expected that the designed antibody models would differ from the naturally occurring ones.

### Benchmark test of the IPRO design

We next assessed the performance of the computational affinity maturation protocol in IPRO using two experimentally characterized GL and AM antibody pairs against influenza virus (AM antibody CH65) and HIV-1 (AM antibody VRC01), respectively. Structures for each pair of GL and AM structures were available [16], [17], [45], [46]. Experimental data shows that the GL precursor of CH65 possesses 200-fold lower binding affinity to HA1 than the matured CH65 [16], and that VRC01 exhibits no detectable affinity for wild-type gp120 [17]. Two design tests were performed. The first one considered mutations only among the residues that differ between GL and AM and a second where all residues could be mutated. The first test aimed to evaluate whether IPRO could redesign the antibody model to match the amino acid usage patterns in the AM pair starting from the GL sequence. The second test, without specified mutation positions, broadly models affinity maturations aiming to evaluate whether IPRO would independently discover results highly similar to natural affinity maturation.

In the first test, forward and reverse designs were conducted for both systems. Forward design is denoted here as a design starting from a GL structure, while reverse design is the one starting from an AM structure. For each design, mutations along the entire sequence were specified according to their corresponding GL/AM partners. For each design, 25 independent IPRO trajectories were generated and the final IE results are the average of the trajectories, as listed in Table 2. As expected, the IEs indicate that the AM antibodies bind to antigens more tightly than the GL antibodies, which is in agreement with the free energy calculation (ΔG) in Table S4. That this result could be recapitulated in forward and reverse designs for both systems demonstrates the robustness of the sampling and scoring function of IPRO. This is critical for the correct identification of a set of mutations that improve binding to an antigen.

**Table 2:** Forward and reverse designs using IPRO with specified positions for mutations.

Antibody	Structure^a^	Mutations^b^		IE^c^		IE difference^d^
		VH	VL	GL	AM	
Influenza CH65	4HK0 (GL)	11	6	−294	−444	−150
	3SM5 (AM)			−295	−389	−94
HIV VRC01	4JPK (GL)	39	25	−167	−258	−90
	3NGB (AM)			−272	−384	−111

a The starting X-ray structures used for IPRO designs. GL in the parentheses indicate the structure is germline and AM is for affinity maturation.

b The number of mutations identified by comparing GL and AM antibody sequences.

c Interaction energy between an antigen and an antibody. All energies are in kcal/mol.

d The IE difference calculated from AM - GL.

In the second test, only forward designs were performed because the aim is to assess the ability of IPRO to recapitulate the native mutations. The preferred amino acid types for FR residues were assigned according to site-specific amino acid probabilities obtained from the alignments of existing broadly neutralizing influenza and HIV antibodies, respectively (Data S1 and S2). No mutation positions or preferred amino acid types for the CDRs were specified. As seen in Table 3, in the designed sequences 35% and 20% of mutations in the native AM influenza and HIV-1 antibody models, respectively, are recaptured. These percentages are comparable to those from a protein interface design for Ran GTPase with 22–39% native sequence recovery using single-constraint design strategy and mutating only interface residues [47]. Our design is more challenging because the entire sequence space requires sampling, typically more than 200 positions for paired heavy and light variable domains. In addition, it has been observed that native somatic mutations evolved from the same GL antibody against the same antigen epitope can be quite diverse [43], [44], [48]. Among the ten VRC01-like HIV broadly neutralizing antibodies isolated by RSC3 binding from different donors, only two residues from the same germline IGHV1-2*02 allele mature to the same amino acids [48]. Therefore, for a given mutation position there exist multiple native mutations. In this test, we used only one AM antibody as the reference for defining the native mutations. The two reported percentages of native mutation recovery would be higher if multiple AM antibodies were used as native references.

**Table 3:** Forward designs using IPRO without specified positions for mutations.

Antibody	Structures^a^	Mutations^b^	Native mutations recovery^c^	IE^d^		IE difference^e^
				GL	AM	
Influenza CH65	4HK0	17	6	−294	−1663	−1359
HIV VRC01	4JPK	64	13	−167	−1247	−1080

a The starting structures used for IPRO designs. GL in the parentheses indicate the structure is germline and AM is for affinity maturation.

b The number of mutations identified by comparing GL and AM antibody sequences.

c The numbers of designed mutations occurring both in GL and AM antibodies.

d Interaction energy between an antigen and an antibody. All energies are in kcal/mol.

e The IE difference calculated from AM - GL.

### Benchmark test for the humanness score

The fundamental assumption of our humanization protocol is that human sequences contain ensembles of local sequences that possess minimal immunogenicity due to a lack of binding to MHC and/or recognition by T cells. Based on this assumption, HScore in OptMAVEn was developed to measure the 9-mer differences of all possible 9-mer frames in a test sequence by comparing them to a precompiled human 9-mer-sequence database. An immunological fragment with size of 9 is chosen because it is the basic peptide unit required for high affinity binding to MHC II [49]. Essentially, an antibody humanness score should have the ability to differentiate human antibody sequences from other species' antibody sequences (e.g. mouse) with high specificity. To meet this end, analysis of the human, mouse, rat and rabbit antibody sequences was performed according to the HScore definition and the results are summarized in Table 4. According to its definition, a lower HScore indicates sequences that are more homologous to human antibody sequences and are predicted to have lower immunogenicity than a higher HScore and vice versa.

**Table 4:** Calculated HScores of human, mouse, rat and rabbit sequences.

Species	Region	Number of sequences	Mean^a^	SD (±)^b^
Human	VH	3000	0	0
	VL	3000	0	0
Synthetic	VH	720	40	41
	VL	599	21	33
Mouse	VH	3462	54	29
	VL	3480	34	27
Rat	VH	1062	57	42
	VL	173	99	49
Rabbit	VH	244	149	18
	VL	244	109	19

a The mean of HScore.

b The standard deviation of HScore.

Overall, there was a marked difference in HScores between the human and mouse sequences with p<0.000001. Even better statistical significances were observed for the rat and rabbit antibodies, but the number of sequences evaluated was many fewer than for mouse. It appears that synthetic sequences are more similar to those of human than mouse, rat and rabbit. For example, synthetic VL has a mean lowest mean HScore of 21. The lower HScore of synthetic antibodies are consistent with their sequence contents, which are often created through joining two genes, one of which is human. Conversely, rabbit VH has the highest mean HScore of 149, indicating it has the most different local 9-mer sequences to human VL sequences. Overall, our results demonstrate that HScore is an efficient humanization score for evaluating the predicted immunogenicity of a target antibody sequence by correctly differentiating human sequences from other mammal.

### Two case studies: Design of models against influenza hemagglutinin and HIV gp120

We showed that OptMAVEn could accurately position antigens in the predefined antibody-binding site, select appropriate antibody parts from MAPs and identify interaction energy improving mutations while distinguishing human from other mammal sequence. OptMAVEn is carried out by combining antigen positioning, MILP based antibody model assembly and IPRO based *in silico* affinity maturation as a complete pipeline. For demonstration we applied OptMAVEn to design broadly neutralizing antibody models that target HA and gp120, which are well-know antigens for the influenza [45], [50]–[52] and HIV-1 [46], [53]–[55] viruses, respectively.

### Identification of epitope

HA proteins, which attach the influenza virus to sialic acid receptors on the cell surface, are a prime drug target. The majority of human antibodies are directed against sites on the head of HA1, in particular the receptor-binding site, to prevent the attachment of virus to target cells. Other antibodies target the stem region to prevent membrane fusion. In this study, we target the receptor-binding site of HA1, the epitope in PDB 3SM5. The B chain and some residues of chain A far from the binding site were ignored to reduce computation time. The receptor-binding site is a broad and shallow pocket framed by three loops and one helix forming the outer ridges, illustrated as the 130-loop (magenta), the 150-loop (yellow), the 190-helix (blue) and the 220-loop (red) in Fig. S3A. The second study involves designing antibody models against the gp120 protein of the human immunodeficiency virus type 1 (HIV-1). Gp120 is a glycoprotein exposed on the surface of the HIV envelope vital for virus entry into a cell. A series of broadly anti-HIV-1 neutralizing antibodies bind to sites located in variable regions of gp120 and help prevent HIV infection. In this study, we designed antibody models targeting the CD4-binding site of gp120 [56]. The gp120 structure was obtained from PDB 3NGB and the epitopes residues are located in CD4-binding-loop, V5-loop and D-loop (Fig. S3B). For each antigen, we chose two targeted epitopes: one includes all the residues in the receptor-binding site (named as HA-all and gp120-all, respectively) and another includes residues that form a loop in the receptor-binding site (i.e., 130-loop for HA1 (HA-130) and CD4-binding-loop for gp120 (gp120–365)).

### Assembly of antibody models from MAPs

**Figure 4:**
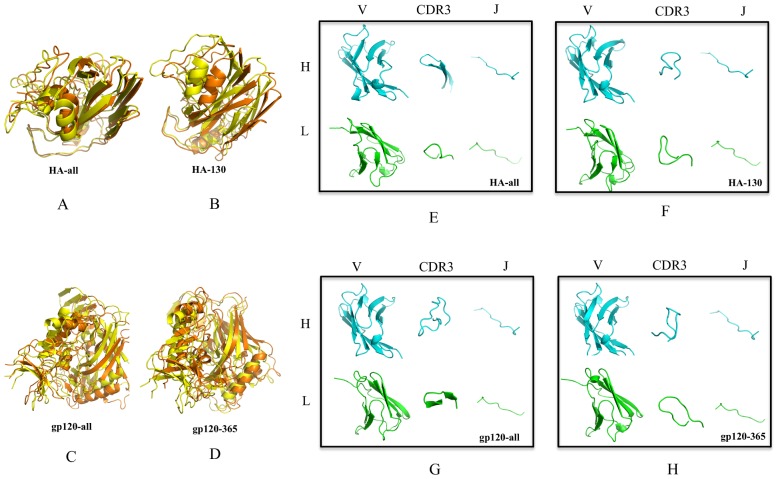
Designed antibody model. (A)–(D) Model structures for epitopes of HA-all, HA-130, gp120-all and gp120–365 before (in yellow) and after (in orange) refinements. (E)–(H) MILP reselected best scored MAPs after refinements. H and L chains are colored in cyan and green, respectively. V, CDR3 and J represent corresponding MAPs (See definition in Method).

Table 5 reports the selected best MAPs structures for each epitope before and after a local conformation refinement of the antigen. The negative MILP energies in each case suggest the selected MAPs structures favor antigen binding. Significantly reduced MILP energies were observed after a refinement of the local antigen conformation and a reselection of the best MAPs structures. Note that the refinement step was only performed for the antigen position with the best-scored MAPs structures and aims to provide a further local conformational sampling around the best conformation from the initial screen. Comparing the antigen conformations before and after refinement (Fig 4A–D), with all RMSDs higher than 2 Å, confirms that local antigen refinement had a pronounced effect on the antigen conformations and the selection of the best-scored MAPs structures.

**Table 5:** Summary of best scored MAPs, RMSDs and MILP energies of the four best designed antibody models for epitopes of HA-all, HA-130, gp120-all, and gp120–365.

Epitope^a^	Refinement^b^	H chain			L chain^c^			Rmsd^d^	MILP^e^
		V	CDR3	J	V	CDR3	J		
HA-all	No	3	58	1	44 (K)	185 (K)	1 (K)	−	−147
	Yes	114	428	5	61 (K)	147 (K)	3 (K)	2.2	−510
HA-130	No	123	283	1	44 (K)	46 (K)	1 (K)	-	−263
	Yes	48	124	1	45 (K)	199 (K)	1 (K)	2.1	−638
gp120-all	No	79	420	5	15 (K)	64 (K)	1 (K)	-	−448
	Yes	79	420	1	27 (K)	7 (L)	1 (L)	2.3	−625
gp120–365	No	19	114	3	2 (K)	1 (K)	3 (K)	-	−402
	Yes	19	196	1	31 (L)	32 (L)	5 (L)	2.1	−736

a The epitope used for designs. See the definition in the method.

b Whether antigen refinement was performed.

c K and L in the parenthesis represent KAMPA and LAMBDA light chains, respectively.

d The Rmsd between the refined and non-refined antigen structures.

e MILP interaction energies. Unit in kcal/mol.

Figure 4E-H show the selected MAPs structures. It can be seen that the CDR3s in both VH and VL exhibit considerable diversity, in accordance with their critical roles in recognizing different epitopes. In addition, the V parts exhibit some variability as well, especially in the CDRs. By contrast, there is less variability in the selected J parts. The selection results from these four designs indicate that the MILP could identify promising initial antigen positions. Once the best low energy conformations are identified, an additional refinement step is required to explore the local antigen positions for obtaining the final modular parts.

### *In silico* affinity maturation and humanization

**Figure 5:**
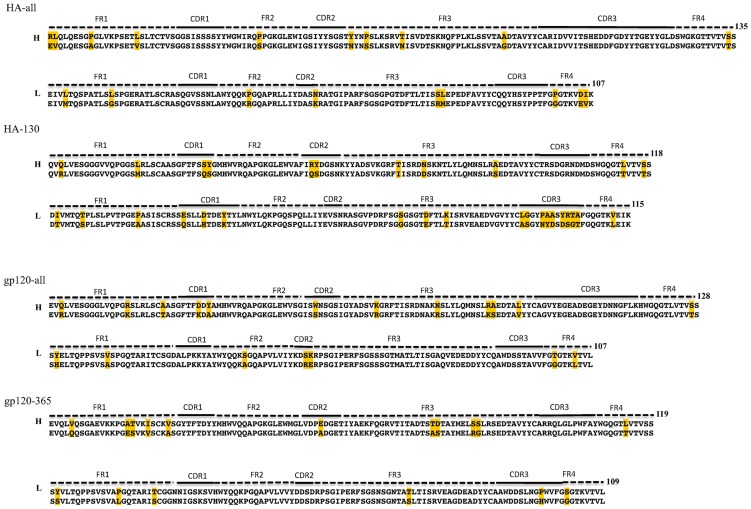
Alignments between designed and initial antibody model sequences for epitopes of HA-all, HA-130, gp120-all and gp120–365. FRs, CDRs regions and the lengths of sequences are indicated on top of each alignment. Yellow shading shows introduced amino acid mutations.

To increase the relevance of the identified designs, the permitted amino acid mutations at each FR position were pre-selected according to the amino acid frequency of each kind of amino acid at that position in alignments of broadly neutralizing HIV and influenza antibodies (Data S1 and S2). However, the residues in CDRs were allowed to mutate into any of the 20 standard amino acids. Figure 5 depicts the predicted lowest-energy amino acid sequences and corresponding alignments between the initial and designed sequences. The sequence distribution of mutations involved in affinity maturation is very diverse, with no clearly preferred amino acids/positions.

The HA-all and HA-130 antibody models have 10/9 and 11/19 mutations in VH/VL, respectively (Table 6). In contrast, gp120-all and gp120–365 antibody models have 12/7 and 11/6 mutations in the corresponding domains. Antibodies typically accumulate 5–20% changes in somatic mutations, but in the case of HIV-1-neutralizing antibodies, the level of somatic mutation ranges from 15% to 44% [57]. Our results suggest that the mutation rate from computational affinity maturation is comparable to that of somatic mutations evolved *in vivo*.

**Table 6:** Summary of energies, mutations and HScores of the four best designed antibody models for epitopes of HA-all, HA-130, gp120-all, and gp120–365.

Epitope^a^	Stage^b^	Complex Energy^c^	IE^d^	Mutation count^e^		HScore^f^	
				H	L	H	L
HA-all	Before	−10588	−184	-	-	6	5
	After	−12391	−1495	10	9	5	5
HA-130	Before	−12413	−99	-	-	0	54
	After	−13965	−1422	11	19	0	32
gp120-all	Before	−14956	−131	-	-	5	7
	After	−15316	−898	12	7	4	3
gp120–365	Before	−14188	−233	-	-	19	4
	After	−14438	−1106	11	6	19	0

a The epitope used for designs. See the definition in the method.

b Before or after the design.

c The entire complex energy. Unit in kcal/mol.

d The interaction energy between the antibody and antigen. Unit in kcal/mol.

e The number of mutations between the designed sequence and the initial sequence.

f The humanness score. See the definition in the method.

**Figure 6:**
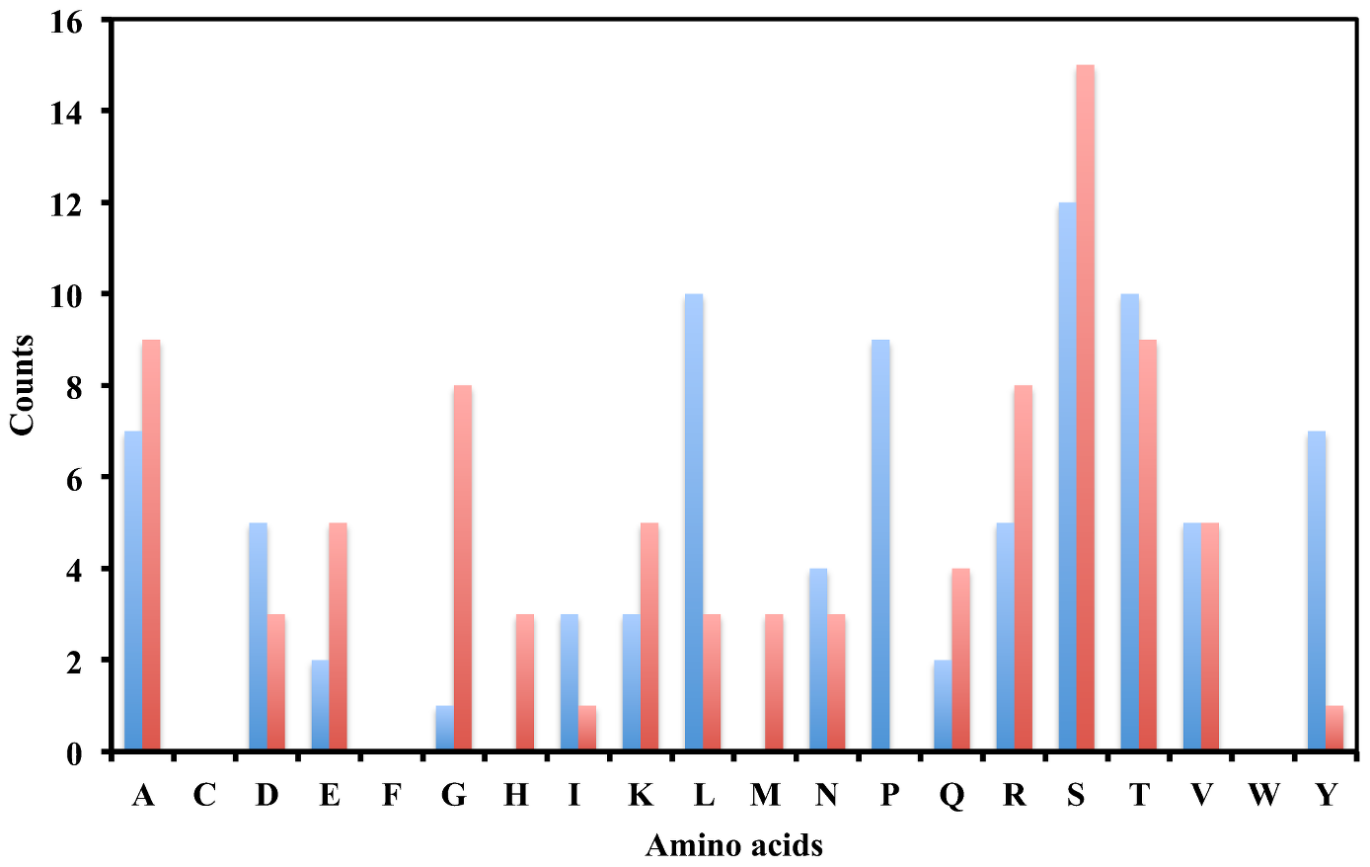
Counts of the type of amino acid mutations before (blue) and after (red) computational affinity maturation from the four best designed antibody models for epitopes of HA-all, HA-130, gp120-all, and gp120–365.

For all four designs, the IPRO results show significant energy improvements both in complex and interaction energies, alluding that the designed antibody models are much more stable and could bind to the antigens more tightly (Table 6). Figure 6 shows the amino acid compositions before and after affinity maturation. Interestingly, Glu, Gly, His, Met, Arg and Ser occur more than other amino acids (counts > = 3) in designed sequences, whereas Leu, Pro and Tyr occur less frequently (counts < = 3). Table 7 summarizes the changes of usage during affinity maturation. An apparent trend is that charged residues are favorable in the affinity mature sequences while aromatic residues are disfavored. Despite the importance of aromatic residues in the binding to antigens [31], this finding is in agreement with previous studies that tyrosine and tryptophan are abundant in the germline genes and the degree of aromaticity is typically reduced during affinity maturation [58]. Meanwhile, the number of polar residues is slightly increased. More polar and charged residue occurrences contribute to the improvement of binding affinity and complex stability in the solvent.

**Table 7:** Statistics of amino acid propensities from the four best designed antibody models for epitopes of HA-all, HA-130, gp120-all and gp120–365.

Stage^a^	Aliphatic^b^	Aromatic^b^	Polar^c^	Charged^c^
Before	35	7	28	15
After	29	1	31	24

a Before or after the design.

b The counts of amino acids belonging to this classification.

Table 6 lists the humanness score for the initial and designed antibody model sequences. Most of the initial sequences, except for the HA-130 light chain and gp120–365 heavy chain possess low humanness scores, indicating they are already human-like sequences and probably have low immunogenicity. The predicted immunogenicity contributions of designed sequences are primarily from CDR3 because the V* and J* model structures in the MAPs database are based on human genes whereas CDR3 has some mouse genes to ensure the maximum amount of structural diversity [25]. After the design, as expected, the humanness scores are decreased or maintained at a similar level. For example, the HScore of gp120–365 light chain is reduced to 0 from 4 and that of HA-130 heavy chain is maintained at 0, both predicting no immunogenicity in the final designs. However, it is also noted that the light chain of HA-130 still has a HScore of 32, comparable to a mouse or rat sequence. This shows that upon design the highest binding affinity design may not have low immunogenicity.

**Figure 7:**
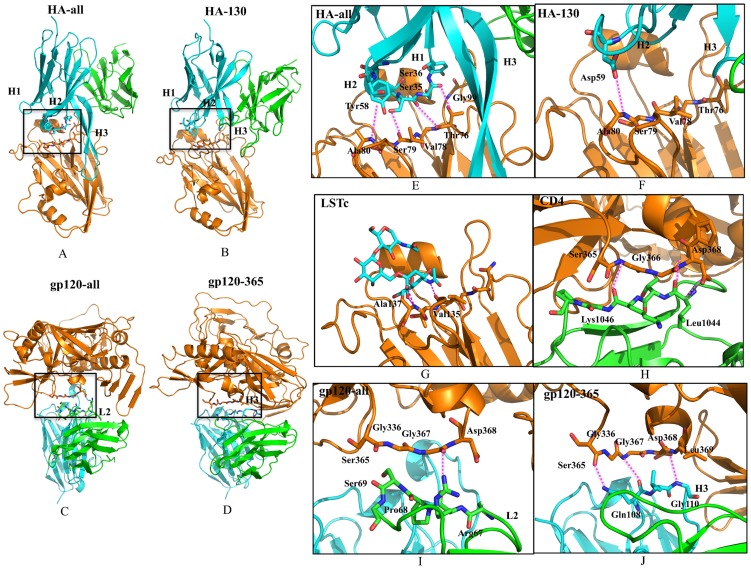
Structures and binding modes of designed antibody models for epitopes of HA-all, HA-130, gp120-all, and gp120–365. H and L chains are colored in cyan and green, respectively. Antigens are colored in orange. Hydrogen bonds are highlighted in dashed line and colored in magenta. (A)–(D) Overall complex structures. (E) and (F) Antibody models that recognize 130-loop in the receptor-binding site of HA1. (G) Interaction of receptor analog LSTc in the receptor-binding site of HA1. (H) Interaction of CD4 and CD4-binding loop of gp120. (I) and (J) Antibody models that recognize of CD4-binding loop of gp120.

The two generated designs against HA1 exhibit highly diverse amino acid sequences, and antigen locations/orientations, but all share the receptor-binding site (Fig. 7A and 7B). Interestingly, the HA-all antibody model possesses a long HCDR3 loop composed of 26 amino acids, which does not bind directly to the epitope residues, but interacts with non-epitope loops on the right side of HA1, as shown in Figure 7A. Such unusually long CDR3s [59] are rarely found in existing HA1 antibodies, and provide a larger antigen-binding surface, potentially introducing more favorable interactions. Moreover, both HCDR1 and HCDR2 insert into the receptor site and interact directly with the 130-loop with four hydrogen bonds (Fig. 7E). In receptor analog LSTc bound to 1934 HA (PDB 1RVZ), the carboxylate group of sialic acid forms two hydrogen bonds with backbone amide of HA1 Ala137, as does the side-chain hydroxyl oxygen of HCDR2 Tyr58 with the backbone amide of HA1 Ala80. The amide of the acetamido group has a hydrogen bond with the backbone oxygen of HCDR1 Val135, as does the backbone oxygen of HA1 with HCDR1 Ser35 (Fig. 7G). In addition, the side-chain carboxyl oxygen of HCDR2 Asp59 of HA-130 antibody model forms the same hydrogen bond with the backbone amide of HA1 Ala80 (Fig. 7F). Thus, the designed HA-all and HA-130 antibody models partially mimic the human receptor that interacts with HA1 via insertion of HCDR1 and HCDR2 into the receptor-binding site. This mode of receptor mimicry has also been observed in related broadly neutralizing antibodies CH65 [45] and 5J8 [60], which use HCDR3 insertion, and S139/1, which uses HCDR2 insertion. Significantly, the algorithm automatically generated these results with the only user input being the identification of the epitope as a whole. The recapitulation of this trend suggests that OptMAVEn could reproduce expected antigen binding motifs. The newly designed antibody models are expected to broadly neutralize a large number of strains from a single HA or selected strains from different subtypes and groups of influenza because the epitope in the receptor-bing site is relatively conserved [61].

Both of the designed antibody models against gp120 are predicted to interact directly with the targeted CD4-binding loop. However, they adopt quite different binding modes (Fig. 7C and 7D). The gp120-all antibody model uses the residues where LCDR2 attaches to grasp virtually all surface-exposed portions of the CD4-binding loop. One hydrogen bond is formed: the side-chain guanidinium nitrogen of Arg67 with the backbone oxygen of gp120 Gly228 (Fig 7I). By contrast, the gp120–365 antibody model uses HCDR3 to interact with CD4-binding loop, which involves three hydrogen bonds: the amide nitrogen of HCDR3 Gln108 with the backbone oxygen of gp120 Ser365; the backbone oxygen of Gln108 with the backbone nitrogen of gp120 Gly367; the backbone nitrogen of HCDR3 Gly110 with the carboxyl oxygen of gp120 Asp368 (Fig. 7J). Among CD4-binding site-directed antibodies, most antibodies (such as the VRC01 class) structurally mimic the CD4 receptor (Fig. 7H, PDB 3JWD) by substantial β-strand contacts to the epitope using HCDR2 [56]. The HCDR3-dominated mode of interaction adopted by the gp120–365 antibody model is similar to a recent isolated broadly neutralizing HIV-1 antibody CH103 [33], which is less mutated than most other CD4-binding site antibodies and reveals a new loop-based mechanism of antibody neutralization.

## Summary and Discussion

Previously, we have developed OptCDR for the *de novo* design of antibody binding pockets composed of CDRs against any specified antigen. We used OptCDR to generate a library of antibody CDRs models against the FLAG antigen (i.e. DYKD) [62] and a preliminary analysis of biological experiments has shown multiple, unique antibody bindings (unpublished data). Here, by expanding the idea of OptCDR, we presented an efficient and general method for *de novo* design of the computational models of the entire antibody variable domain targeting a specific antigen epitope. Given an antigen, the method entails positioning the antigen in a virtual antibody-binding site, identifying the best modular parts from the MAPs database based on the positioned antigen poses, *de novo* assembly of these MAPs into the initial antibody model structure, and designing mutations in the antibody model structure to simultaneously improve binding affinity and reduce immunogenicity. For both HA and gp120, novel antibody models with numerous interactions with their target epitopes were generated. The observed rates of mutations and types of amino acid changes are consistent with what has been seen during *in vivo* affinity maturation. It is especially encouraging that these features were not controlled for in OptMAVEn but were automatically generated. Additionally, in the case of the HA antibody models, a method of antigen binding that has been previously observed in broadly neutralizing anti-influenza antibodies was discovered in both designs.

In OptMAVEn, a pre-generated conformer library was adopted to represent the possible antigen binding poses. Despite success in most of the 120 benchmark cases (success rate of 96%), in some cases, the antigen positioning algorithm still could not sample any conformation having a RMSD within 4 Å of the native. This limitation may have largely been due to the insufficient rotational sampling of antigens around the X and Y axes. Although this limitation could be overcome by adding further sampling around both axes, caution should be taken. For each antigen conformation, its IE to each modular antibody part needs to be pre-calculated and this computation could quickly become intractable with a significantly increased number of antigen conformations. Therefore, it is suggested to choose an appropriate size of the conformer library by adjusting the parameters of antigen positioning if further sampling is required.

OptMAVEn generates a prototype antibody model that targets an antigen by assembling the six best-scored MAPs structures. This idea is inspired by the natural evolution of an antibody *in vivo,* in that the gene of a germline antibody is initially assembled by V-(D)-J recombination. Note that the three differing MAPs regions do not match exactly with their V-(D)-J genes because MAPs utilizes CDR3 as a structural component representing the whole diversity region. The primary advantage of using MAPs to assemble an antibody model is that the prediction is fast and there is no need for *de novo* folding calculations. The computational savings do not come at the expense of accuracy of prediction, as the RMSD of the predicted structures is at least as accurate as earlier methods [25]. In addition, compared to traditional fragment-assembly-based approaches for *de novo* protein structure prediction, this approach could efficiently sample both local and non-local contacts that are inherently present in the relatively large structural fragments. The designed sequences therefore should have a higher probability of proper folding. It should be also noted that MAPs database includes a considerable amount of information on pairwise clashes between parts. These clashes can thus be avoided by not selecting both members of the pair by the MILP problem in OptMAVEn. The aim of the MILP problem is to find the best combination of the six MAPs with the lowest IEs with the targeted antigen. In its current implementation, it does not explicitly account for the interactions between MAPs except for excluding clashing pairs. In our benchmark test, we found that the selected MAPs have better IEs than native antibodies in 42.5% of 120 cases. While approximations in the energy function may account for some of the improved scores, the general trends allude that the cohort of designed sequences should bind more tightly than the native ones.

A simplification of the protocol in its described implementation is that it uses IE instead of binding free energy for evaluating the binding affinity of an antigen and antibody model. Although we found that IEs are in good agreement with experimental binding data in two GL-AM antibody pairs, this replacement is still at the expense of an overall bias in energy function, especially in systems where conformational entropy is crucial for the binding interaction. This is supported by the fact that we obtained large negative IEs (Table S3) that are not quantitatively comparable to true experimental binding energies for predicting the binding affinity of native antigens and antibodies. This may have been due to a lack of entropy term in the formulation of IE (Table S4). Effective entropy could quantify the variability of sequence and their rotamer states that are consistent with a target structure and other properties specified as constraints on the properties of the sequences [63]. However, the role of side-chain entropy plays in protein design is still unclear. For example, both Kendra [17] and Daniele *et al*. [64] demonstrate that changes in protein conformational entropy can contribute significantly to protein sequence design. Conversely, Hu *et al*. suggest that side-chain conformational entropy has a relatively small role in determining the preferred amino acid at each residue position in a protein except for longer amino acids: methionine and arginine [17]. Nevertheless, future work will consider introducing an appropriate entropy model into the energy evaluation.

Another concern in the energy function is the employed Lazaridis-Karplus implicit solvation model [65] which does not adequately capture charged residue interactions with the solvent. This is reflected by some test designs where charged residues were continually selected without solvent exposure (data not shown). This is a limitation of current implicit solvation models, which yield higher or similar water-to-protein transfer free energies for nonpolar as for many of the polar residues. As a result, they improperly favor the burial of polar amino acids in the protein interior over nonpolar ones [66].

During design runs, we also found that the energy calculations often end up with a local energy minimum instead of the global energy minimum due to the incomplete sampling of the complex energy landscape given the allotted cpu time. This could lead to inconsistent results between different repetitions of the same computational design run. To address this issue, we introduced ensemble structure refinement, which is a refinement carried out by generating an ensemble of structures (default of 10) from the “refinement” iterations (default of 25) of IPRO. A structural refinement has the same steps as a normal IPRO iteration except no mutations are allowed. After all refinement iterations have been completed, the average properties of the refinement ensemble are evaluated to determine whether or not a particular design is actually the best identified so far. This ensemble approach to evaluating structures has given a high correlation (R^2^ = 0.960) between calculated IEs and experimentally measured binding affinities in previous work [17].

Another challenge for the *de novo* design is the uncertainty associated with the employed scoring function [63]. For example, energy functions are approximate, side-chain conformations are treated discretely and solvation is represented using simplified models. One solution is to apply site-specific amino acid probabilities, which takes advantage of known inter-atomic interactions and structural features to yield sequence information consistent with naturally folded structures and desired functional properties. It is well known that highly conserved residues are generally distributed in the FRs, whereas the residues in CDRs tend to be considerably diverse. Therefore, in OptMAVEn, we applied two different strategies to determine the permitted amino acid kinds to mutate for residues both from FRs and CDRs. The permitted kinds for FRs residues were site-specific and the probability in each position was obtained from the sequence alignment of broadly neutralizing HIV and influenza antibodies (Data S1 and S2), while those for CDRs residues had no any constraint at all. In both case studies, multiple solutions with quite different sequences and binding modes were identified. Improved mutations were found in both the FRs and the CDRs. The designed antibody models have an apparent trend of obtaining more polar and charged residues while disfavoring aromatic residues.

Designed antibody models could be immunogenic and cause an unexpected and serious immune reaction if applied. To address this issue, we introduced an HScore to evaluate the immunogenicity of designed antibody models *in silico*. The aim is to limit the sequence design to mutations that either maintain or increase the human-like sequence content. Our algorithm is inspired by a very similar humanization method called HSC [23], which is calculated by determining the maximal identity between a string of test sequence and any sequence in an aligned set of human sequences, and summing theses values over all pertinent sequence positions. Although HScore works well to distinguish human antibody sequences from other species, it should be noted that OptMAVEn does not guarantee the design of antibody models without any immunogenicity (HScore  = 0) under the current design parameters. OptMAVEn seeks to design antibody variant models simultaneously having reduced immunogenicity and improved binding *in silico*, and these two properties are often in inverse proportion (i.e. high affinity binders may possess high immunogenicity and vice versa). This is supported by our case studies, where the designed light chain of HA-130 still has an HScore of 32. This is comparable to what would be expected in a mouse antibody. Also, fewer mutations were found to occur in CDR3, where a considerable amount of energetically favorable mutations were observed in naturally occurring AM antibodies. Parker *et al.* demonstrated a similar difficulty in optimizing both the stability and immunogenicity of therapeutic proteins by a structure-guided deimmunization strategy [28]. Another probable reason for the failure of deimmunization is that the maximum number of allowed residues (3 under the current study) to mutate and the site-specific amino acid probabilities both impose limits on the number of sequences for immunogenicity evaluation. During some IPRO iterations, few or no mutations are presented for further energy optimization. As a result, in general trade-offs must be made to design antibody models either with predicted moderate binding affinity and lowest immunogenicity or highest binding affinity and moderate immunogenicity.

## Supporting Information

Figure S1 **The assembly and affinity maturation of gemline antibody.** The variable region of the heavy chain is generated from variable (V), diversity (D) and joining (J) gene segments, whereas the variable regions of the light chains are generated from V and J gene segments. For the heavy chain, the first two CDRs and three framework regions (FRs) of the variable region are encoded by V gene. CDR3 is encoded by a few nucleotides of V, all of D, and part of J segment, while FR4 is encoded by the remainder of the J segment. For the light chain, V gene segment encode the first two CDRs and three FRs of the V region, plus a few residues of CDR3. J segment encodes the remainder of CDR3 and the fourth FR.
(TIF)

Figure S2 **The distribution of the mean XYZ coordinates of antigen epitopes.** (A) Along X axis. (B) Along Y axis (C) Along Z axis.
(TIF)

Figure S3 **The epitopes of influenza HA1 (A) and HIV-1 gp120 (B).** H and L chains are colored in cyan and green, respectively. Antigens are colored in yellow and epitopes are colored in blue, magenta and yellow, respectively.
(TIF)

Table S1 **Numbers of antibody modular part structures in MAPs.**
(DOCX)

Table S2 **Native antibody-antigen structure set.**
(DOCX)

Table S3 **Interaction energies for antibody-antigen complexes.**
(DOCX)

Table S4 **Binding free energy for forward and reverse designs using IPRO with specified positions for mutations.**
(DOCX)

Data S1 **The alignments of broadly neutralizing HIV and influenza antibody sequences.**
(TXT)

Data S2 **The alignments of broadly neutralizing HIV and influenza antibody sequences.**
(TXT)

## References

[1] Beck A, Wurch T, Bailly C, Corvaia N (2010) Strategies and challenges for the next generation of therapeutic antibodies. Nat Rev Immunol 10: 345–352. 20414207 10.1038/nri2747

[2] Nelson AL, Reichert JM (2009) Development trends for therapeutic antibody fragments. Nat Biotechnol 27: 331–337. 19352366 10.1038/nbt0409-331

[3] Carter PJ (2006) Potent antibody therapeutics by design. Nat Rev Immunol 6: 343–357. 16622479 10.1038/nri1837

[4] Morrison SL, Johnson MJ, Herzenberg LA, Oi VT (1984) Chimeric human antibody molecules: mouse antigen-binding domains with human constant region domains. Proc Natl Acad Sci U S A 81: 6851–6855. 6436822 10.1073/pnas.81.21.6851 PMC392030

[5] Boulianne GL, Hozumi N, Shulman MJ (1984) Production of functional chimaeric mouse/human antibody. Nature 312: 643–646. 6095115 10.1038/312643a0

[6] Jackel C, Kast P, Hilvert D (2008) Protein design by directed evolution. Annu Rev Biophys 37: 153–173. 18573077 10.1146/annurev.biophys.37.032807.125832

[7] Hoogenboom HR (2005) Selecting and screening recombinant antibody libraries. Nat Biotechnol 23: 1105–1116. 16151404 10.1038/nbt1126

[8] Smith GP (1985) Filamentous fusion phage: novel expression vectors that display cloned antigens on the virion surface. Science 228: 1315–1317. 4001944 10.1126/science.4001944

[9] Hoess RH (2001) Protein design and phage display. Chem Rev 101: 3205–3218. 11710069 10.1021/cr000056b

[10] Brenke R, Hall DR, Chuang GY, Comeau SR, Bohnuud T, et al (2012) Application of asymmetric statistical potentials to antibody-protein docking. Bioinformatics 28: 2608–2614. 23053206 10.1093/bioinformatics/bts493 PMC3467743

[11] Chaudhury S, Berrondo M, Weitzner BD, Muthu P, Bergman H, et al (2011) Benchmarking and analysis of protein docking performance in Rosetta v3.2. PLoS One 6: e22477. 21829626 10.1371/journal.pone.0022477 PMC3149062

[12] Krawczyk K, Baker T, Shi J, Deane CM (2013) Antibody i-Patch prediction of the antibody binding site improves rigid local antibody-antigen docking. Protein Eng Des Sel 26: 621–629. 24006373 10.1093/protein/gzt043

[13] Simonelli L, Pedotti M, Beltramello M, Livoti E, Calzolai L, et al (2013) Rational engineering of a human anti-dengue antibody through experimentally validated computational docking. PLoS One 8: e55561. 23405171 10.1371/journal.pone.0055561 PMC3566030

[14] Sellers BD, Nilmeier JP, Jacobson MP (2010) Antibodies as a model system for comparative model refinement. Proteins 78: 2490–2505. 20602354 10.1002/prot.22757 PMC2998178

[15] Sivasubramanian A, Sircar A, Chaudhury S, Gray JJ (2009) Toward high-resolution homology modeling of antibody Fv regions and application to antibody-antigen docking. Proteins 74: 497–514. 19062174 10.1002/prot.22309 PMC2909601

[16] Li T, Tracka MB, Uddin S, Casas-Finet J, Jacobs DJ, et al (2014) Redistribution of Flexibility in Stabilizing Antibody Fragment Mutants Follows Le Chatelier's Principle. PLoS One 9: e92870. 24671209 10.1371/journal.pone.0092870 PMC3966838

[17] Li T, Verma D, Tracka MB, Casas-Finet J, Livesay DR, et al.. (2013) Thermodynamic Stability and Flexibility Characteristics of Antibody Fragment Complexes. Protein Pept Lett. 10.2174/09298665113209990051 PMC4667953 23855672

[18] Lippow SM, Wittrup KD, Tidor B (2007) Computational design of antibody-affinity improvement beyond in vivo maturation. Nat Biotechnol 25: 1171–1176. 17891135 10.1038/nbt1336 PMC2803018

[19] Barderas R, Desmet J, Timmerman P, Meloen R, Casal JI (2008) Affinity maturation of antibodies assisted by in silico modeling. Proc Natl Acad Sci U S A 105: 9029–9034. 18574150 10.1073/pnas.0801221105 PMC2449359

[20] Clark LA, Boriack-Sjodin PA, Eldredge J, Fitch C, Friedman B, et al (2006) Affinity enhancement of an in vivo matured therapeutic antibody using structure-based computational design. Protein Sci 15: 949–960. 16597831 10.1110/ps.052030506 PMC2242497

[21] Pantazes RJ, Maranas CD (2010) OptCDR: a general computational method for the design of antibody complementarity determining regions for targeted epitope binding. Protein Eng Des Sel 23: 849–858. 20847101 10.1093/protein/gzq061

[22] Yu CM, Peng HP, Chen IC, Lee YC, Chen JB, et al (2012) Rationalization and design of the complementarity determining region sequences in an antibody-antigen recognition interface. PLoS One 7: e33340. 22457753 10.1371/journal.pone.0033340 PMC3310866

[23] Lazar GA, Desjarlais JR, Jacinto J, Karki S, Hammond PW (2007) A molecular immunology approach to antibody humanization and functional optimization. Mol Immunol 44: 1986–1998. 17079018 10.1016/j.molimm.2006.09.029

[24] Zhang D, Chen CF, Zhao BB, Gong LL, Jin WJ, et al (2013) A novel antibody humanization method based on epitopes scanning and molecular dynamics simulation. PLoS One 8: e80636. 24278299 10.1371/journal.pone.0080636 PMC3836750

[25] Pantazes RJ, Maranas CD (2013) MAPs: a database of modular antibody parts for predicting tertiary structures and designing affinity matured antibodies. BMC Bioinformatics 14: 168. 23718826 10.1186/1471-2105-14-168 PMC3687570

[26] Saraf MC, Moore GL, Goodey NM, Cao VY, Benkovic SJ, et al (2006) IPRO: an iterative computational protein library redesign and optimization procedure. Biophys J 90: 4167–4180. 16513775 10.1529/biophysj.105.079277 PMC1459523

[27] Nielsen M, Lundegaard C, Worning P, Lauemoller SL, Lamberth K, et al (2003) Reliable prediction of T-cell epitopes using neural networks with novel sequence representations. Protein Sci 12: 1007–1017. 12717023 10.1110/ps.0239403 PMC2323871

[28] Parker AS, Choi Y, Griswold KE, Bailey-Kellogg C (2013) Structure-guided deimmunization of therapeutic proteins. J Comput Biol 20: 152–165. 23384000 10.1089/cmb.2012.0251 PMC3576912

[29] Jardine J, Julien JP, Menis S, Ota T, Kalyuzhniy O, et al (2013) Rational HIV immunogen design to target specific germline B cell receptors. Science 340: 711–716. 23539181 10.1126/science.1234150 PMC3689846

[30] Schmidt AG, Xu H, Khan AR, O'Donnell T, Khurana S, et al (2013) Preconfiguration of the antigen-binding site during affinity maturation of a broadly neutralizing influenza virus antibody. Proc Natl Acad Sci U S A 110: 264–269. 23175789 10.1073/pnas.1218256109 PMC3538208

[31] Livesay DR, Subramaniam S (2004) Conserved sequence and structure association motifs in antibody-protein and antibody-hapten complexes. Protein Eng Des Sel 17: 463–472. 15310840 10.1093/protein/gzh058

[32] Jespers LS, Roberts A, Mahler SM, Winter G, Hoogenboom HR (1994) Guiding the selection of human antibodies from phage display repertoires to a single epitope of an antigen. Biotechnology (N Y) 12: 899–903. 7521646 10.1038/nbt0994-899

[33] Liao HX, Lynch R, Zhou T, Gao F, Alam SM, et al (2013) Co-evolution of a broadly neutralizing HIV-1 antibody and founder virus. Nature 496: 469–476. 23552890 10.1038/nature12053 PMC3637846

[34] Abhinandan KR, Martin AC (2007) Analyzing the “degree of humanness” of antibody sequences. J Mol Biol 369: 852–862. 17442342 10.1016/j.jmb.2007.02.100

[35] Hwang WY, Foote J (2005) Immunogenicity of engineered antibodies. Methods 36: 3–10. 15848070 10.1016/j.ymeth.2005.01.001

[36] Gao SH, Huang K, Tu H, Adler AS (2013) Monoclonal antibody humanness score and its applications. BMC Biotechnol 13: 55. 23826749 10.1186/1472-6750-13-55 PMC3729710

[37] Vanommeslaeghe K, Hatcher E, Acharya C, Kundu S, Zhong S, et al (2010) CHARMM general force field: A force field for drug-like molecules compatible with the CHARMM all-atom additive biological force fields. J Comput Chem 31: 671–690. 19575467 10.1002/jcc.21367 PMC2888302

[38] Ehrenmann F, Giudicelli V, Duroux P, Lefranc MP (2011) IMGT/Collier de Perles: IMGT standardized representation of domains (IG, TR, and IgSF variable and constant domains, MH and MhSF groove domains). Cold Spring Harb Protoc 2011: 726–736. 21632776 10.1101/pdb.prot5635

[39] Lefranc MP (2011) IMGT unique numbering for the variable (V), constant (C), and groove (G) domains of IG, TR, MH, IgSF, and MhSF. Cold Spring Harb Protoc 2011: 633–642. 21632789 10.1101/pdb.ip85

[40] Lefranc MP (2011) IMGT Collier de Perles for the variable (V), constant (C), and groove (G) domains of IG, TR, MH, IgSF, and MhSF. Cold Spring Harb Protoc 2011: 643–651. 21632788 10.1101/pdb.ip86

[41] Lefranc MP, Pommie C, Kaas Q, Duprat E, Bosc N, et al (2005) IMGT unique numbering for immunoglobulin and T cell receptor constant domains and Ig superfamily C-like domains. Dev Comp Immunol 29: 185–203. 15572068 10.1016/j.dci.2004.07.003

[42] ILOG (2013) ILOG CPLEX 12.4 User's Manual. ILOG Inc, Mountain View, CA, USA.

[43] Willis JR, Briney BS, DeLuca SL, Crowe JE Jr, Meiler J (2013) Human germline antibody gene segments encode polyspecific antibodies. PLoS Comput Biol 9: e1003045. 23637590 10.1371/journal.pcbi.1003045 PMC3636087

[44] Yin J, Beuscher AEt, Andryski SE, Stevens RC, Schultz PG (2003) Structural plasticity and the evolution of antibody affinity and specificity. J Mol Biol 330: 651–656. 12850137 10.1016/s0022-2836(03)00631-4

[45] Whittle JR, Zhang R, Khurana S, King LR, Manischewitz J, et al (2011) Broadly neutralizing human antibody that recognizes the receptor-binding pocket of influenza virus hemagglutinin. Proc Natl Acad Sci U S A 108: 14216–14221. 21825125 10.1073/pnas.1111497108 PMC3161572

[46] Zhou T, Georgiev I, Wu X, Yang ZY, Dai K, et al (2010) Structural basis for broad and potent neutralization of HIV-1 by antibody VRC01. Science 329: 811–817. 20616231 10.1126/science.1192819 PMC2981354

[47] Humphris EL, Kortemme T (2007) Design of multi-specificity in protein interfaces. PLoS Comput Biol 3: e164. 17722975 10.1371/journal.pcbi.0030164 PMC1950952

[48] James LC, Roversi P, Tawfik DS (2003) Antibody multispecificity mediated by conformational diversity. Science 299: 1362–1367. 12610298 10.1126/science.1079731

[49] Reche PA, Reinherz EL (2003) Sequence variability analysis of human class I and class II MHC molecules: functional and structural correlates of amino acid polymorphisms. J Mol Biol 331: 623–641. 12899833 10.1016/s0022-2836(03)00750-2

[50] Fleishman SJ, Whitehead TA, Ekiert DC, Dreyfus C, Corn JE, et al (2011) Computational design of proteins targeting the conserved stem region of influenza hemagglutinin. Science 332: 816–821. 21566186 10.1126/science.1202617 PMC3164876

[51] Lingwood D, McTamney PM, Yassine HM, Whittle JR, Guo X, et al (2012) Structural and genetic basis for development of broadly neutralizing influenza antibodies. Nature 489: 566–570. 22932267 10.1038/nature11371 PMC7095019

[52] Xu R, Ekiert DC, Krause JC, Hai R, Crowe JE Jr, et al (2010) Structural basis of preexisting immunity to the 2009 H1N1 pandemic influenza virus. Science 328: 357–360. 20339031 10.1126/science.1186430 PMC2897825

[53] Walker LM, Huber M, Doores KJ, Falkowska E, Pejchal R, et al (2011) Broad neutralization coverage of HIV by multiple highly potent antibodies. Nature 477: 466–470. 21849977 10.1038/nature10373 PMC3393110

[54] Walker LM, Phogat SK, Chan-Hui PY, Wagner D, Phung P, et al (2009) Broad and potent neutralizing antibodies from an African donor reveal a new HIV-1 vaccine target. Science 326: 285–289. 19729618 10.1126/science.1178746 PMC3335270

[55] Miller BR, Demarest SJ, Lugovskoy A, Huang F, Wu X, et al (2010) Stability engineering of scFvs for the development of bispecific and multivalent antibodies. Protein Eng Des Sel 23: 549–557. 20457695 10.1093/protein/gzq028

[56] Kwong PD, Mascola JR, Nabel GJ (2013) Broadly neutralizing antibodies and the search for an HIV-1 vaccine: the end of the beginning. Nat Rev Immunol 13: 693–701. 23969737 10.1038/nri3516

[57] Corti D, Lanzavecchia A (2013) Broadly neutralizing antiviral antibodies. Annu Rev Immunol 31: 705–742. 23330954 10.1146/annurev-immunol-032712-095916

[58] Burkovitz A, Sela-Culang I, Ofran Y (2014) Large-scale analysis of somatic hypermutations in antibodies reveals which structural regions, positions and amino acids are modified to improve affinity. FEBS J 281: 306–319. 24279419 10.1111/febs.12597

[59] Wu TT, Johnson G, Kabat EA (1993) Length distribution of CDRH3 in antibodies. Proteins 16: 1–7. 8497480 10.1002/prot.340160102

[60] Hong M, Lee PS, Hoffman RM, Zhu X, Krause JC, et al (2013) Antibody recognition of the pandemic H1N1 Influenza virus hemagglutinin receptor binding site. J Virol 87: 12471–12480. 24027321 10.1128/JVI.01388-13 PMC3807900

[61] Xu R, Krause JC, McBride R, Paulson JC, Crowe JE Jr, et al (2013) A recurring motif for antibody recognition of the receptor-binding site of influenza hemagglutinin. Nat Struct Mol Biol 20: 363–370. 23396351 10.1038/nsmb.2500 PMC3594569

[62] Einhauer A, Jungbauer A (2001) The FLAG peptide, a versatile fusion tag for the purification of recombinant proteins. J Biochem Biophys Methods 49: 455–465. 11694294 10.1016/s0165-022x(01)00213-5

[63] Schneider G (2013) De novo molecular design. John Wiley & Sons, Inc: 480.

[64] Sciretti D, Bruscolini P, Pelizzola A, Pretti M, Jaramillo A (2009) Computational protein design with side-chain conformational entropy. Proteins 74: 176–191. 18618711 10.1002/prot.22145

[65] Lazaridis T, Karplus M (1999) Effective energy function for proteins in solution. Proteins 35: 133–152. 10223287 10.1002/(sici)1097-0134(19990501)35:2<133::aid-prot1>3.0.co;2-n

[66] Jaramillo A, Wodak SJ (2005) Computational protein design is a challenge for implicit solvation models. Biophys J 88: 156–171. 15377512 10.1529/biophysj.104.042044 PMC1304995

