# Supplementary figures and images for: OptMAVEn – A New Framework for the de novo Design of Antibody Variable Region Models Targeting Specific Antigen Epitopes
Source: PLoS One. 2014 Aug 25;9(8):e105954. doi: 10.1371/journal.pone.0105954 (PMC4143332; doi:10.1371/journal.pone.0105954)

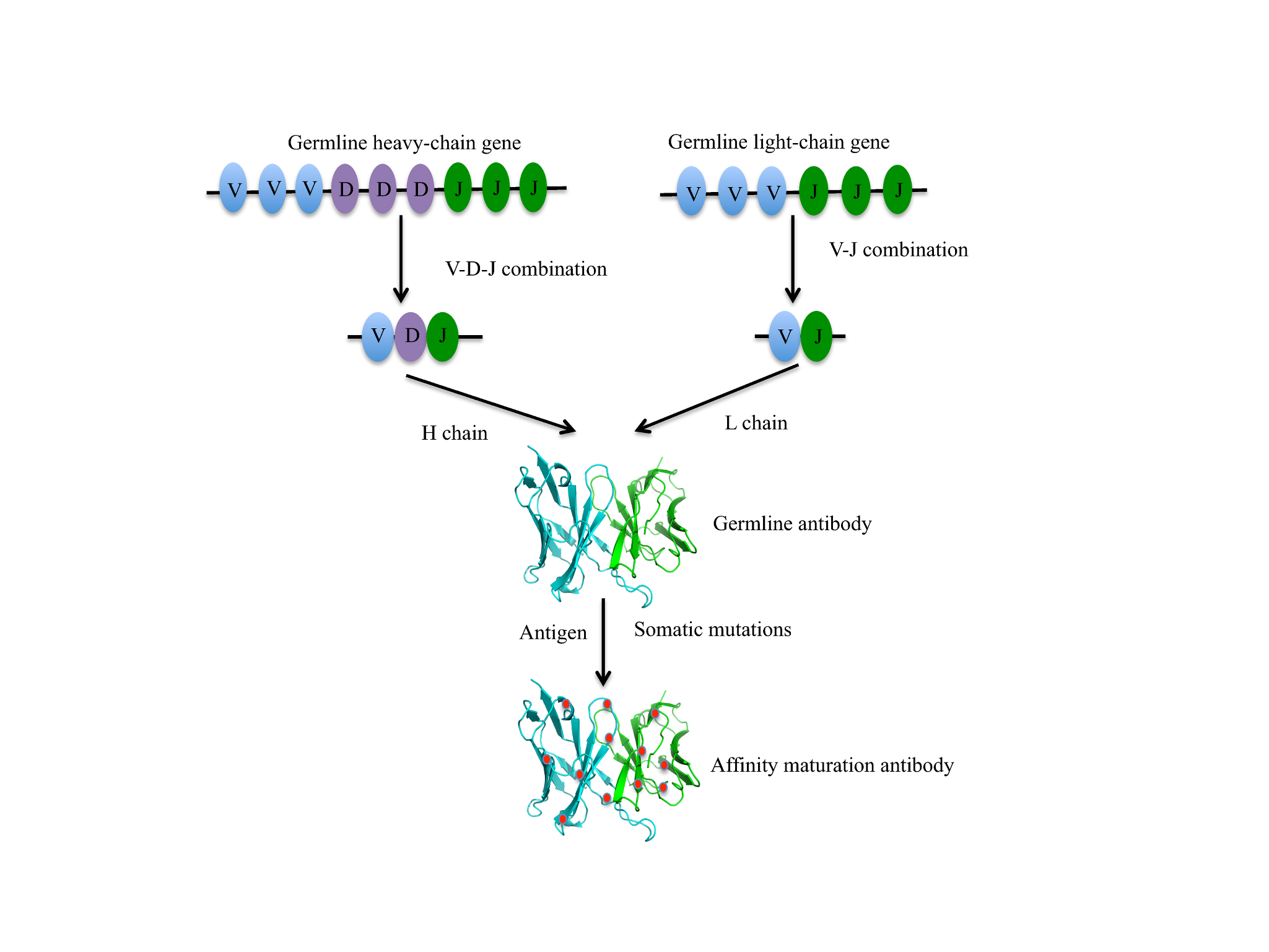

Supplement: Figure S1 — The assembly and affinity maturation of gemline antibody. The variable region of the heavy chain is generated from variable (V), diversity (D) and joining (J) gene segments, whereas the variable regions of the light chains are generated from V and J gene segments. For the heavy chain, the first two CDRs and three framework regions (FRs) of the variable region are encoded by V gene. CDR3 is encoded by a few nucleotides of V, all of D, and part of J segment, while FR4 is encoded by the remainder of the J segment. For the light chain, V gene segment encode the first two CDRs and three FRs of the V region, plus a few residues of CDR3. J segment encodes the remainder of CDR3 and the fourth FR. (TIF) [file pone.0105954.s001.tif]

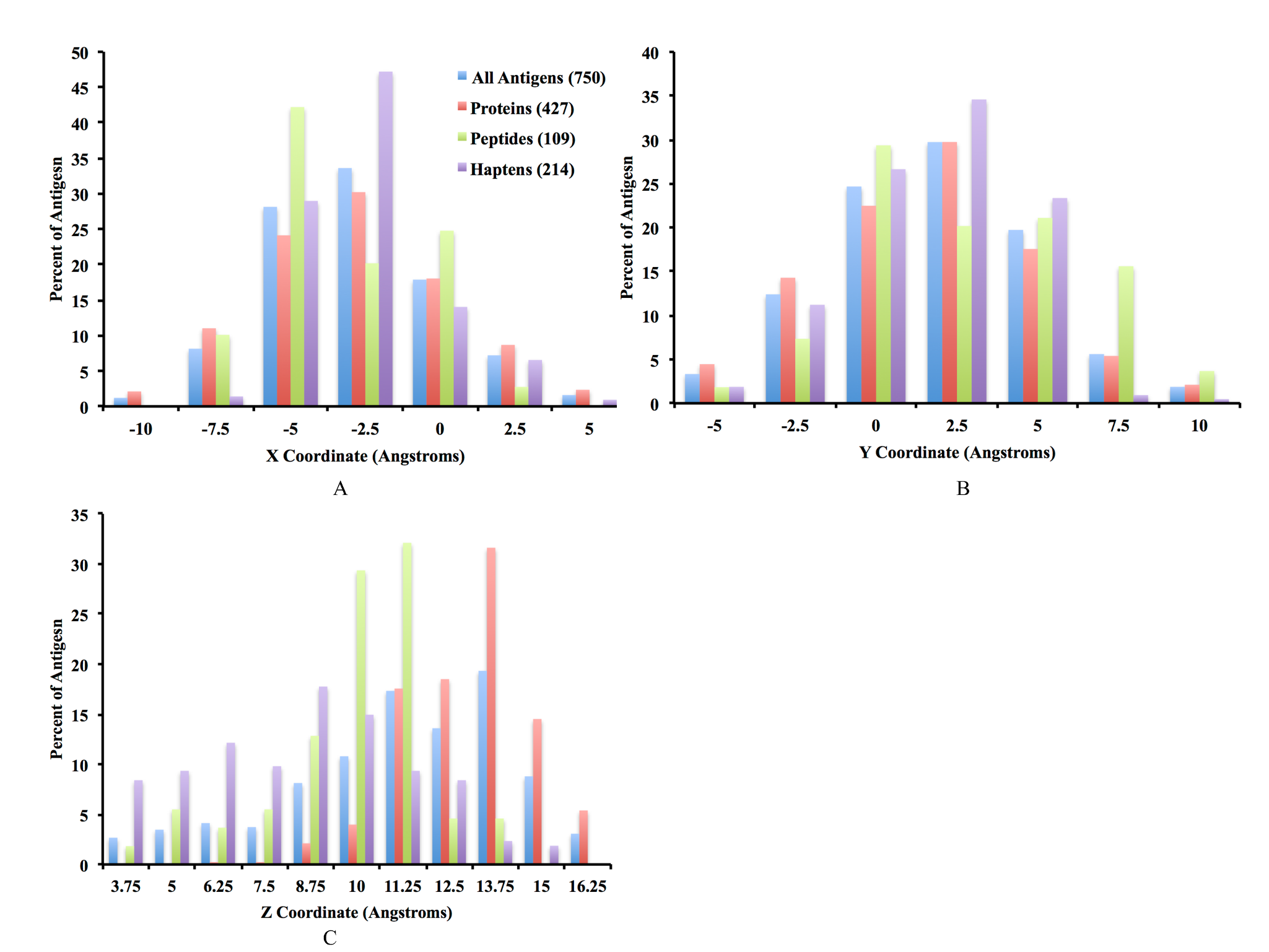

Supplement: Figure S2 — The distribution of the mean XYZ coordinates of antigen epitopes. (A) Along X axis. (B) Along Y axis (C) Along Z axis. (TIF) [file pone.0105954.s002.tif]

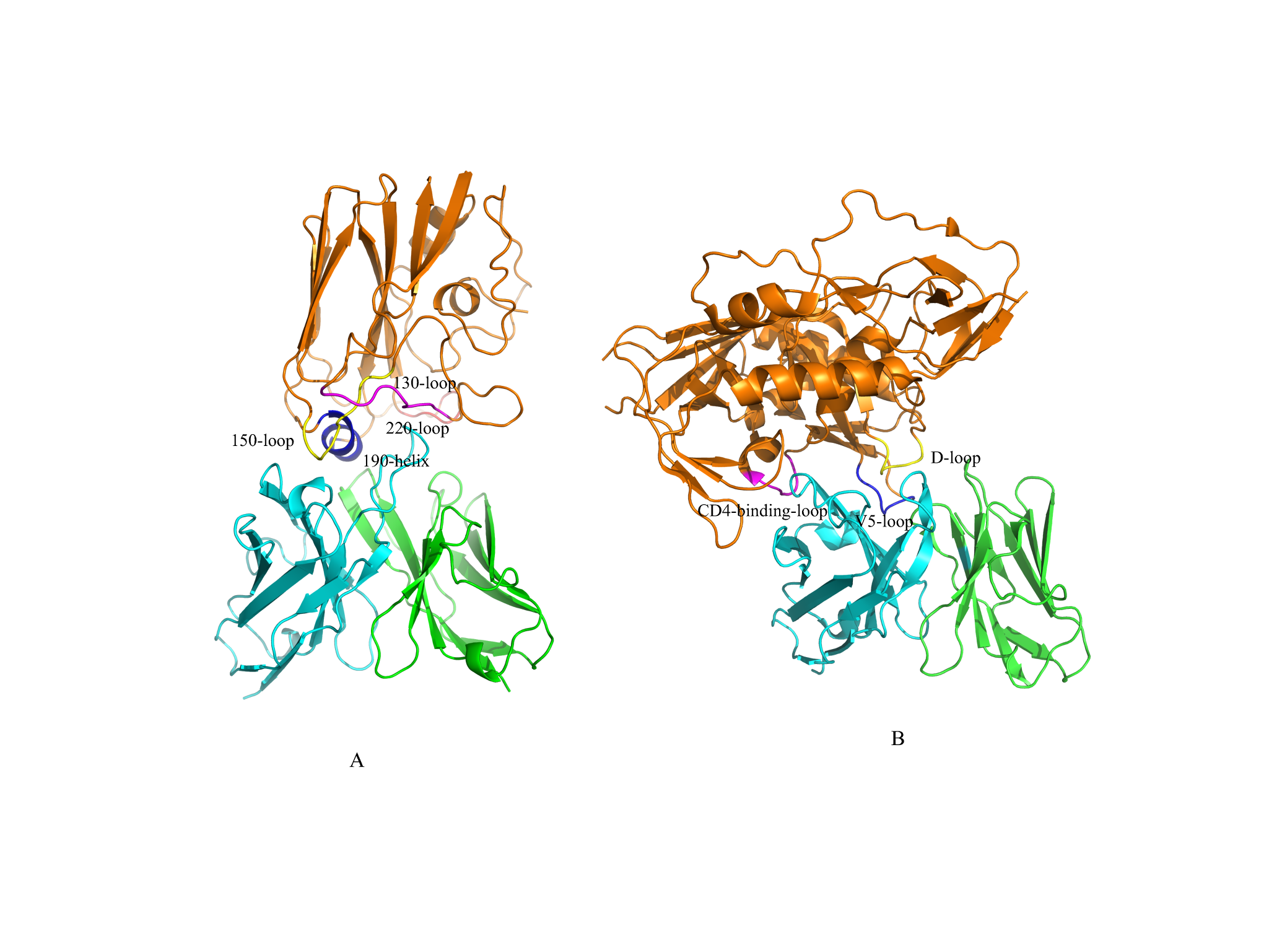

Supplement: Figure S3 — The epitopes of influenza HA1 (A) and HIV-1 gp120 (B). H and L chains are colored in cyan and green, respectively. Antigens are colored in yellow and epitopes are colored in blue, magenta and yellow, respectively. (TIF) [file pone.0105954.s003.tif]
