# Supplementary material for: OptMAVEn – A New Framework for the de novo Design of Antibody Variable Region Models Targeting Specific Antigen Epitopes
Source: PLoS One. 2014 Aug 25;9(8):e105954. doi: 10.1371/journal.pone.0105954 (PMC4143332; doi:10.1371/journal.pone.0105954)
Supplement: Table S1 — Numbers of antibody modular part structures in MAPs. (DOCX) [file pone.0105954.s004.docx]

Table S1. Numbers of antibody modular part structures in MAPs

|  | H chain | L chain | |
| --- | --- | --- | --- |
|  |  | KAPPA | LAMBDA |
| V^a^ | 141 | 67 | 38 |
| CDR3^b^ | 428 | 199 | 39 |
| J^c^ | 5 | 5 | 7 |
| Antibodies | 3 × 10^5^ | 6.7 × 10^4^ | 1 × 10^4^ |
| Total Antibodies | 2.3 × 10^10^ | | |

^a^ Variable region. ^b^ Diversity region. ^c^ Joining region
