# Supplementary material for: OptMAVEn – A New Framework for the de novo Design of Antibody Variable Region Models Targeting Specific Antigen Epitopes
Source: PLoS One. 2014 Aug 25;9(8):e105954. doi: 10.1371/journal.pone.0105954 (PMC4143332; doi:10.1371/journal.pone.0105954)
Supplement: Table S2 — Native antibody-antigen structure set. (DOCX) [file pone.0105954.s005.docx]

Table S2. Native antibody-antigen structure set

| PDB | Length | Antigen | Epitope |
| --- | --- | --- | --- |
| 2OR9 | 11 | cMyc | 1 2 3 4 5 6 7 8 9 10 |
| 3QG6 | 6 | agr | 1 2 3 4 5 6 |
| 2J4W | 34 | ama1 | 7 8 11 12 13 14 15 16 17 18 30 31 32 33 |
| 2R0W | 7 | amyloid | 1 2 3 4 5 6 7 |
| 3BAE | 7 | amyloid | 1 2 3 4 5 6 7 |
| 2CK0 | 11 | angiotensin | 1 3 4 5 6 7 8 9 10 11 |
| 3ETB | 143 | anthrax | 58 60 61 62 63 64 66 89 90 91 92 93 94 95 96 125 127 |
| 2G5B | 7 | bax | 1 2 3 4 5 6 7 |
| 3AB0 | 136 | bcla | 23 25 34 40 41 42 43 44 46 75 77 78 79 107 109 111 113 |
| 3NH7 | 85 | bmpr-1a | 10 27 29 31 32 34 41 44 45 46 47 48 49 51 53 57 58 59 60 61 62 63 64 |
| 3E8U | 11 | bnp | 1 2 3 4 5 6 7 8 10 |
| 3RKD | 146 | capsid | 18 19 21 26 27 38 39 40 41 50 52 54 55 56 57 76 114 115 116 117 118 119 120 134 |
| 2HKF | 9 | carbonic | 1 2 3 4 5 6 7 8 9 |
| 2OSL | 20 | cd20 | 2 3 4 5 6 7 8 9 10 |
| 3BKY | 17 | cd20 | 2 3 4 5 6 7 8 9 |
| 1XIW | 85 | cd3e | 38 40 41 45B 45C 45D 45E 45F 45G 78 82 83 105 107 108 112 113 114 115 116 |
| 3LQA | 98 | cd4 | 74 75 77 78 79 80 81 92 93 |
| 3O2D | 99 | cd4 | 10 44 49 71 96 97 98 99 100 122 124 |
| 1I9R | 143 | cd40 | 11 12 14 23 24 25 26 28 60 61 99 100 102 127 128 129 130 131 133 |
| 1CE1 | 8 | cd52 | 1 2 3 4 5 6 7 8 |
| 1TET | 12 | cholera | 1 2 3 4 5 6 7 8 9 10 11 |
| 2H1P | 11 | cryptococcus | 1 2 3 4 5 7 8 9 10 11 |
| 1E4W | 7 | cyclic | 1 2 3 4 5 6 7 |
| 3KS0 | 92 | cytochrome | 25 58 59 60 61 62 63 64 65 67 69 |
| 1ZA3 | 91 | death | 5 6 7 14 16 17 18 33 34 36 37 38 39 41 42 |
| 2A6I | 9 | dodecapeptide | 2 3 4 5 6 7 8 9 |
| 2ZUQ | 148 | dsbb | 85 86 87 88 89 90 91 92 118 119 120 127 |
| 3G5V | 16 | egfr | 1 2 3 7 10 11 12 13 14 15 16 |
| 1I8I | 9 | egfr | 1 2 4 5 6 7 8 |
| 1ZTX | 101 | envelope | 3 7 8 9 10 29 31 32 33 34 66 67 68 69 90 91 92 |
| 1EJO | 13 | fmdv | 1 2 3 4 5 6 7 8 9 11 12 |
| 1P4B | 12 | gcn4 | 2 3 4 5 6 7 9 10 |
| 2QHR | 11 | gp | 1 2 3 4 5 6 7 8 9 10 11 |
| 1ACY | 10 | gp120 | 2 3 4 5 6 7 8 |
| 2QSC | 15 | gp120 | 5 6 7 8 9 10 11 12 13 |
| 3F58 | 11 | gp120 | 1 2 3 4 5 6 7 8 9 10 |
| 3GHE | 15 | gp120 | 1 2 3 4 5 6 7 8 9 10 11 |
| 3MLR | 14 | gp120 | 1 2 3 4 5 6 7 8 9 11 13 14 |
| 3MLW | 17 | gp120 | 1 2 3 4 5 6 7 8 9 11 13 14 |
| 1GGI | 9 | gp120 | 2 3 4 5 6 7 8 |
| 3MLX | 14 | gp120 | 1 2 3 4 5 6 7 8 9 10 11 12 13 |
| 2B1H | 16 | gp120 | 1 2 3 4 5 6 7 8 9 13 14 |
| 3MLY | 12 | gp120 | 1 2 3 4 5 6 7 8 9 10 11 12 |
| 3GHB | 10 | gp120 | 1 2 3 4 5 6 7 8 9 |
| 1QNZ | 18 | gp120 | 2 3 4 5 6 7 8 9 10 11 12 13 14 17 |
| 2OQJ | 18 | gp120 | 1 2 3 8 10 11 12 13 14 15 |
| 3MLS | 20 | gp120 | 3 5 6 10 11 12 13 14 15 16 17 18 |
| 1TZG | 12 | gp160 | 2 3 4 5 6 7 8 9 12 |
| 3FN0 | 9 | gp160 | 1 2 3 4 5 6 7 |
| 1U8J | 7 | gp41 | 1 2 3 4 5 6 7 |
| 3P30 | 84 | gp41 | 56 59 63 66 67 69 70 73 74 77 |
| 1OBE | 13 | gp41 | 1 2 3 4 5 6 7 8 9 10 11 12 |
| 1HIN | 8 | hemagglutinin | 1 2 3 4 5 6 7 8 |
| 2EH8 | 10 | hepatitis | 1 2 3 4 5 6 7 8 9 10 |
| 1W72 | 90 | hla | 65 66 68 69 72 73 75 76 79 80 83 84 86 89 |
| 2JEL | 85 | hpr | 1 2 3 4 34 36 41 64 66 67 68 70 71 72 75 76 |
| 3L5W | 101 | il-13 | 5 8 9 92 95 96 98 99 100 |
| 3L5Y | 85 | il-13 | 2 6 9 10 78 79 81 82 83 |
| 3G6D | 106 | il-13 | 2 3 11 15 16 18 19 20 21 94 97 98 101 102 104 105 106 |
| 3IU3 | 119 | il-2 | 1 2 3 4 5 6 22 23 25 27 28 29 36 39 40 41 42 43 45 46 47 48 56 57 79 81 |
| 3NFP | 124 | il-2 | 1 2 3 4 5 6 25 27 39 41 76 78 80 109 112 113 114 115 |
| 1EGJ | 101 | il-5 | 25 26 27 28 29 30 58 79 80 81 82 84 |
| 1JRH | 95 | interferon | 37 39 40 41 42 43 44 45 46 66 68 69 70 72 74 88 89 |
| 2VXS | 80 | interleukin | 37 48 49 50 51 56 76 77 78 79 |
| 3D85 | 133 | interleukin | 59 63 64 65 68 69 70 71 72 83 87 98 99 100 101 102 104 105 |
| 2HVK | 103 | k chanel 1 | 24 27 28 31 32 33 34 35 36 37 39 40 41 42 43 |
| 3PJS | 139 | k chanel 1 | 118 121 125 126 128 129 132 133 136 137 139 |
| 1ORS | 132 | k chanel 2 | 88 92 93 94 95 96 97 98 100 101 104 |
| 1KB5 | 111 | kb5-c20 | 2 3 27 108 109 110 113 114 115 117 118 |
| 1FBI | 129 | lysozyme | 14 15 16 20 21 62 63 71 72 73 75 77 89 93 96 97 100 101 102 |
| 2DQJ | 129 | lysozyme | 14 15 16 19 20 21 62 63 73 74 75 77 93 96 97 98 100 101 102 103 |
| 1JHL | 129 | lysozyme | 21 22 23 102 103 104 106 111 112 113 116 117 118 119 121 |
| 1KIQ | 129 | lysozyme | 18 19 22 23 24 25 27 102 103 116 117 118 119 120 121 124 125 |
| 1MLC | 129 | lysozyme | 41 43 45 46 47 48 49 50 51 53 66 67 68 70 79 81 84 |
| 2IFF | 129 | lysozyme | 41 43 45 46 47 48 49 51 53 67 68 69 70 81 84 |
| 1DZB | 129 | lysozyme | 20 21 23 62 63 73 75 96 97 98 100 101 102 103 104 106 112 116 |
| 3HR5 | 9 | m1p peptide | 1 2 3 4 5 6 7 8 9 |
| 2BDN | 68 | mcp1 | 25 27 28 29 31 34 35 36 37 38 52 53 58 61 62 65 66 |
| 1UWX | 13 | membrane peptide | 2 3 4 5 6 7 8 11 12 |
| 3QWO | 53 | motavizumab | 1 2 3 4 6 7 10 11 14 24 25 28 29 32 |
| 1NAK | 10 | mp1 | 2 3 4 5 6 7 8 9 |
| 1SM3 | 9 | muc1 | 1 2 3 4 5 6 7 8 9 |
| 2IGF | 7 | myohemerythrin | 1 2 3 4 5 6 7 |
| 3O0R | 142 | nitric oxide reductase | 100 101 102 103 105 141 |
| 1F90 | 9 | nonapeptide | 1 2 3 4 5 6 7 8 |
| 3GJG | 9 | nyeso-1 peptide | 1 2 3 4 5 6 7 8 9 |
| 1PZ5 | 8 | octapeptide | 1 2 3 4 5 6 7 8 |
| 1KTR | 4 | oligohistidine | 1 2 3 4 |
| 3CXD | 9 | osteopontin | 1 2 4 5 6 7 8 9 |
| 3CVH | 8 | ovalbumin | 4 5 6 7 8 |
| 1TQB | 102 | ovprp | 1 2 32 33 59 62 63 64 65 66 67 68 69 70 71 72 73 |
| 1MVU | 13 | p-glycoprotein | 1 2 3 5 6 7 9 10 |
| 1HH6 | 11 | p24 1 | 2 3 4 5 6 7 8 9 10 11 |
| 1CFS | 11 | p24 2 | 1 2 4 5 6 7 8 9 10 11 |
| 1CFT | 5 | p24 3 | 1 2 3 4 5 |
| 2VXQ | 92 | phl p 2 | 28 30 32 37 38 39 41 65 66 67 72 73 74 75 76 77 78 |
| 1FPT | 11 | polio | 4 5 6 7 8 9 10 11 |
| 1QKZ | 10 | porin | 1 2 3 4 5 6 7 8 9 10 |
| 2HH0 | 9 | prion | 1 2 3 4 5 6 7 8 9 |
| 1JP5 | 6 | protease | 1 2 3 4 5 6 |
| 2HRP | 10 | protease | 1 2 3 4 5 6 7 8 9 10 |
| 2ZPK | 8 | protease | 1 2 3 4 5 6 7 8 |
| 1RJL | 95 | protein b | 30 31 32 49 50 51 52 53 71 72 73 74 75 76 |
| 1N64 | 16 | protein c | 1 2 5 6 7 8 9 10 11 12 13 14 |
| 2R29 | 97 | protein e | 9 10 11 12 13 14 15 28 65 66 67 90 91 92 93 94 |
| 1KC5 | 8 | ps1 | 1 2 3 4 5 6 7 |
| 1KCR | 15 | ps1 | 1 2 3 4 5 6 7 8 11 12 13 14 |
| 3FFD | 18 | pth-rp | 1 3 4 6 7 8 10 11 13 14 15 17 18 |
| 1XGY | 6 | rhodopsin | 1 2 3 4 5 6 |
| 1CU4 | 10 | shaprp | 2 3 4 5 6 7 8 9 |
| 3GGW | 11 | shigella | 1 2 4 5 6 8 |
| 1NSN | 138 | snase | 6 54 57 58 61 65 67 90 92 93 94 95 102 103 117 118 120 121 124 |
| 3EO1 | 112 | tgfb | 28 29 30 31 32 90 91 92 93 94 101 |
| 1V7M | 145 | thrombopoietin | 51 52 55 62 65 69 92 95 96 99 100 103 104 105 106 107 108 |
| 3EYU | 6 | tkr | 1 2 3 4 5 6 |
| 2HFG | 27 | tnfr | 11 12 13 14 15 16 17 19 20 21 22 23 24 25 26 |
| 1OAZ | 115 | trx-shear3 | 33 34 35 37 41 42 43 46 48 80 81 82 83 98 99 100 101 102 105 |
| 3DVG | 75 | ubiquitin | 1 20 21 22 24 57 58 59 60 61 62 63 64 |
| 1BJ1 | 94 | vegf 1 | 32 35 66 67 68 69 70 71 73 74 75 76 77 78 79 80 81 |
| 2QR0 | 97 | vegf 1 | 33 36 69 70 71 72 74 75 76 77 78 79 |
| 1TZH | 94 | vegf 1 | 32 33 66 69 70 71 72 73 74 75 76 77 78 79 80 |
| 3BDY | 95 | vegf 1 | 32 35 68 70 71 72 73 74 75 76 77 78 79 80 |
| 1TZI | 97 | vegf 1 | 5 6 51 54 94 |
| 2FJH | 98 | vegf 1 | 5 6 7 8 10 11 12 14 50 55 90 93 |
| 2VWE | 98 | vegf 2 | 7 8 11 15 16 17 52 56 91 92 93 95 |
