# Supplementary material for: OptMAVEn – A New Framework for the de novo Design of Antibody Variable Region Models Targeting Specific Antigen Epitopes
Source: PLoS One. 2014 Aug 25;9(8):e105954. doi: 10.1371/journal.pone.0105954 (PMC4143332; doi:10.1371/journal.pone.0105954)
Supplement: Table S3 — Interaction energies for antibody-antigen complexes. (DOCX) [file pone.0105954.s006.docx]

Table S3. Interaction energies for antibody-antigen complexes.

| Name | Native^a^ | | MAPs^b^ | | Name | Native | | MAPs | |
| --- | --- | --- | --- | --- | --- | --- | --- | --- | --- |
|  | MILP^c^ | Charmm^d^ | MILP | Charmm |  | MILP | Charmm | MILP | Charmm |
| 1ACY | -141 | -206 | -344 | -326 | 2HFG | -129 | -208 | -266 | -217 |
| 1BJ1 | -27 | -180 | -90 | -147 | 2HH0 | -298 | -411 | -355 | -377 |
| 1CE1 | -355 | -429 | -253 | -221 | 2HKF | -397 | -478 | -263 | -283 |
| 1CFS | -194 | -272 | -209 | -260 | 2HRP | -87 | -160 | -271 | -287 |
| 1CFT | -99 | -140 | -319 | -330 | 2HVK | -383 | -505 | -362 | -437 |
| 1CU4 | -394 | -500 | -277 | -386 | 2IFF | 2215 | -399 | -363 | -300 |
| 1DZB | -276 | -408 | -369 | -454 | 2IGF | -231 | -327 | -301 | -355 |
| 1E4W | -19 | -102 | -234 | -248 | 2J4W | -193 | -281 | -330 | -404 |
| 1EGJ | -239 | -304 | -263 | -239 | 2JEL | -122 | -232 | -352 | -354 |
| 1EJO | -176 | -286 | -385 | -337 | 2OQJ | -56 | -129 | -163 | -154 |
| 1F90 | -153 | -193 | -303 | -286 | 2OR9 | -187 | -267 | -113 | -102 |
| 1FBI | -292 | -469 | -258 | -281 | 2OSL | -99 | -157 | -90 | -97 |
| 1FPT | -364 | -474 | -292 | -335 | 2QHR | -353 | -471 | -328 | -330 |
| 1GGI | -252 | -321 | -358 | -268 | 2QR0 | -17 | -99 | -300 | -312 |
| 1HH6 | -117 | -232 | -218 | -175 | 2QSC | -141 | -198 | -313 | -334 |
| 1HIN | -110 | -195 | -192 | -238 | 2R0W | -161 | -240 | -273 | -254 |
| 1I8I | -398 | -474 | -394 | -403 | 2R29 | -198 | -328 | -184 | -206 |
| 1I9R | -104 | -238 | -288 | -325 | 2VWE | -98 | -182 | -113 | -178 |
| 1JHL | -179 | -277 | -330 | -332 | 2VXQ | -280 | -388 | -372 | -441 |
| 1JP5 | -167 | -214 | -179 | -192 | 2VXS | -36 | -92 | -120 | -161 |
| 1JRH | -351 | -464 | -335 | -305 | 2ZPK | -66 | -127 | -143 | -156 |
| 1KC5 | -136 | -188 | -172 | -167 | 2ZUQ | -140 | -213 | -115 | -146 |
| 1KCR | -15 | -97 | -226 | -272 | 3AB0 | -224 | -301 | -407 | -443 |
| 1KIQ | -84 | -171 | -188 | -251 | 3BAE | -92 | -173 | -284 | -307 |
| 1KTR | 50 | -7 | -236 | -216 | 3BDY | -94 | -170 | -247 | -211 |
| 1MLC | -191 | -288 | -230 | -208 | 3BKY | -78 | -131 | -191 | -35 |
| 1MVU | -66 | -116 | -331 | -356 | 3CVH | -134 | -189 | -215 | -153 |
| 1N64 | -114 | -197 | -148 | -192 | 3CXD | -211 | -295 | -271 | -265 |
| 1NAK | -237 | -307 | -227 | -269 | 3D85 | -69 | -181 | -165 | -196 |
| 1NSN | -81 | -210 | -490 | -493 | 3DVG | -90 | -195 | -186 | -222 |
| 1OAZ | 32 | -82 | -217 | -273 | 3E8U | -125 | -200 | -232 | -231 |
| 1OBE | -301 | -373 | -86 | -201 | 3ETB | -461 | -593 | -378 | -410 |
| 1ORS | -151 | -211 | -280 | -297 | 3EYU | -165 | -241 | -320 | -269 |
| 1P4B | -48 | -126 | -146 | -114 | 3F58 | -203 | -286 | -336 | -316 |
| 1PZ5 | -23 | -123 | -122 | -133 | 3FFD | -378 | -512 | -264 | -324 |
| 1QKZ | -75 | -143 | -170 | -192 | 3FN0 | -98 | -176 | -143 | -182 |
| 1QNZ | -148 | -240 | -228 | -322 | 3G5V | -157 | -262 | -230 | -280 |
| 1RJL | -129 | -210 | -208 | -201 | 3G6D | -607 | -729 | -602 | -686 |
| 1SM3 | -67 | -125 | -267 | -278 | 3GGW | -23 | -88 | -194 | -222 |
| 1TET | -152 | -222 | -181 | -159 | 3GHB | -291 | -352 | -111 | -80 |
| 1TQB | -264 | -379 | -364 | -302 | 3GHE | -246 | -387 | -266 | -305 |
| 1TZG | -128 | -220 | -162 | -192 | 3GJG | -6 | -85 | -90 | -114 |
| 1TZH | 21 | -57 | -172 | -201 | 3HR5 | -233 | -340 | -447 | -415 |
| 1TZI | -14 | -37 | -147 | -160 | 3IU3 | -550 | -801 | -110 | -306 |
| 1U8J | -271 | -337 | -297 | -315 | 3KS0 | -58 | -148 | -195 | -248 |
| 1UWX | -65 | -127 | -303 | -305 | 3L5W | -448 | -522 | -497 | -535 |
| 1V7M | -68 | -182 | -193 | -264 | 3L5Y | -422 | -571 | -406 | -476 |
| 1W72 | -158 | -238 | -251 | -274 | 3LQA | -41 | -82 | -210 | -224 |
| 1XGY | -147 | -226 | -353 | -314 | 3MLR | -274 | -345 | -281 | -390 |
| 1XIW | -368 | -478 | -275 | -321 | 3MLS | -290 | -406 | -336 | -402 |
| 1ZTX | -214 | -315 | -410 | -400 | 3MLW | -379 | -490 | -513 | -597 |
| 2A6I | 1888 | -93 | 116 | -191 | 3MLX | -72 | -174 | -132 | -143 |
| 2B1H | -490 | -600 | -286 | -364 | 3MLY | -139 | -246 | -206 | -231 |
| 2BDN | -281 | -407 | -283 | -526 | 3NFP | -165 | -352 | -307 | -415 |
| 2CK0 | -38 | -109 | -111 | -124 | 3NH7 | -620 | -783 | -508 | -569 |
| 2DQJ | -235 | -382 | -272 | -371 | 3O0R | -68 | -120 | -209 | -234 |
| 2EH8 | -178 | -276 | -277 | -210 | 3P30 | -202 | -305 | -244 | -288 |
| 2FJH | -56 | -164 | -345 | -256 | 3QG6 | -28 | -130 | -165 | -183 |
| 2G5B | -109 | -173 | -282 | -257 | 3QWO | -209 | -339 | -495 | -476 |
| 2H1P | -48 | -115 | -172 | -137 | 3RKD | -225 | -348 | -278 | -325 |

^a^Native indicates the energies are calculated using native antigen and antibody from the X-ray structures

^b^MAPs indicates the energies are calculated using native antigen from the X-ray structures and antibody from the MAPs databases

^c^The energy for MILP selection calculated using pairwise energy function including van der Waals and electrostatic terms

^d^The energy calculated using Charmm after minimized the structure including van der Waals, electrostatics, bonds, angles, dihedral angles, improper dihedral angles and generalized Born with molecular volume integration implicit solvation terms.

All energies are in kcal/mol.
