# Supplementary material for: OptMAVEn – A New Framework for the de novo Design of Antibody Variable Region Models Targeting Specific Antigen Epitopes
Source: PLoS One. 2014 Aug 25;9(8):e105954. doi: 10.1371/journal.pone.0105954 (PMC4143332; doi:10.1371/journal.pone.0105954)
Supplement: Table S4 — Binding free energy for forward and reverse designs using IPRO with specified positions for mutations. (DOCX) [file pone.0105954.s007.docx]

Table S4. Binding free energy for forward and reverse designs using IPRO with specified positions for mutations. For all the four cases, the free energies of AM antibodies more favor the binding to the antigens than those of GL antibodies. Meanwhile, the same trend could be observed based on the enthalpy, which is in agreement with our interaction energy results. The effect of entropy is fairly complicated. In general, entropy disfavors the binding. For the HIV VRC01 antibody the binding with GL antibodies is more entropically disfavored than binding with AM antibodies while for influenza CH65 antibodies the reverse trends are observed. However, in both cases the enthalpic contribution dominates the overall free energy values.

| Antibody | Structure^a^ | ΔH ^b^ | | -TΔS^b^ | | | | ΔG | | | | ΔG  difference^d^ |
| --- | --- | --- | --- | --- | --- | --- | --- | --- | --- | --- | --- | --- |
|  |  | GL | AM | GL | AM | | | GL | | AM | |  |
| Influenza CH65 | 4HK0 (GL) | -2 | -3 | 2 | | 0 | 0 | | -3 | | -3 | |
|  | 3SM5 (AM) | -9 | -33 | 6 | | 28 | | -3 | | -5 | | -2 |
| HIV VRC01 | 4JPK (GL) | -76 | -108 | 68 | | 59 | | -18 | | -49 | | -31 |
|  | 3NGB (AM) | -89 | -97 | 64 | | 50 | | -25 | | -47 | | -22 |

^a^ The starting X-ray structures used for IPRO designs. GL in the parentheses indicate the structure is germline and AM is for affinity maturation.

^b^ The enthalpy and entropy between the antigen and antibody calculated by MM-PBSA method using 2 ns molecular dynamic trajectories. Some residues (residue 1-46 and 269-323) in the stem region of hemagglutinin (4HK0 and 3SM5) far from the binding site were removed for reducing the calculation time. Unit is in kcal/mol.

^c^ The free energy ΔG = ΔH - TΔS. Unit is in kcal/mol.

^d^ The ΔG difference calculated from AM - GL.
